# Model Reduction Captures Stochastic Gamma Oscillations on Low-Dimensional Manifolds

**DOI:** 10.3389/fncom.2021.678688

**Published:** 2021-08-17

**Authors:** Yuhang Cai, Tianyi Wu, Louis Tao, Zhuo-Cheng Xiao

**Affiliations:** ^1^Department of Statistics, University of Chicago, Chicago, IL, United States; ^2^School of Mathematical Sciences, Peking University, Beijing, China; ^3^Center for Bioinformatics, National Laboratory of Protein Engineering and Plant Genetic Engineering, School of Life Sciences, Peking University, Beijing, China; ^4^Center for Quantitative Biology, Peking University, Beijing, China; ^5^Courant Institute of Mathematical Sciences, New York University, New York, NY, United States

**Keywords:** gamma oscillations, synchrony, homogeneity, coarse-graining method, model reduction algorithm

## Abstract

Gamma frequency oscillations (25–140 Hz), observed in the neural activities within many brain regions, have long been regarded as a physiological basis underlying many brain functions, such as memory and attention. Among numerous theoretical and computational modeling studies, gamma oscillations have been found in biologically realistic spiking network models of the primary visual cortex. However, due to its high dimensionality and strong non-linearity, it is generally difficult to perform detailed theoretical analysis of the emergent gamma dynamics. Here we propose a suite of Markovian model reduction methods with varying levels of complexity and apply it to spiking network models exhibiting heterogeneous dynamical regimes, ranging from nearly homogeneous firing to strong synchrony in the gamma band. The reduced models not only successfully reproduce gamma oscillations in the full model, but also exhibit the same dynamical features as we vary parameters. Most remarkably, the invariant measure of the coarse-grained Markov process reveals a two-dimensional surface in state space upon which the gamma dynamics mainly resides. Our results suggest that the statistical features of gamma oscillations strongly depend on the subthreshold neuronal distributions. Because of the generality of the Markovian assumptions, our dimensional reduction methods offer a powerful toolbox for theoretical examinations of other complex cortical spatio-temporal behaviors observed in both neurophysiological experiments and numerical simulations.

## 1. Introduction

Modern experimental techniques have revealed a vast diversity of coherent spatiotemporal activity patterns in the brain, reflecting the many possible interactions between excitation and inhibition, between cellular and synaptic time-scales, and between local and long-range circuits. Prominent amongst these patterns are the rich repertoire of neuronal oscillations that can be stimulus driven or internally generated and are likely to be responsible for sensory perception and cognitive tasks (Fries, 2009; Tallon-Baudry, 2009). In particular, gamma band oscillations (25–140 Hz), observed in multi-unit activity (MUA) and local field potential (LFP) measurements (Ray and Maunsell, 2015), have been found in many brain regions (visual cortex (Gray et al., 1989; Azouz and Gray, 2000; Logothetis et al., 2001; Henrie and Shapley, 2005), auditory cortex (Brosch et al., 2002), somatosensory cortex (Bauer et al., 2006), parietal cortex (Pesaran et al., 2002; Buschman and Miller, 2007; Medendorp et al., 2007), frontal cortex (Buschman and Miller, 2007; Gregoriou et al., 2009; Siegel et al., 2009; Sohal et al., 2009; Canolty et al., 2010; Sigurdsson et al., 2010; van Wingerden et al., 2010), hippocampus (Bragin et al., 1995; Csicsvari et al., 2003; Colgin et al., 2009; Colgin, 2016), amygdala (Popescu et al., 2009), and striatum (Van Der Meer and Redish, 2009)). Much experimental evidence correlates gamma oscillations to behavior and enhanced sensory or cognitive performances. For instance, gamma dynamics has been shown to sharpen orientation tuning in V1 and speed and direction tuning in MT (Azouz and Gray, 2000, 2003; Frien et al., 2000; Liu and Newsome, 2006; Womelsdorf et al., 2012). During cognitive tasks, the increases in gamma power in the visual pathway have been shown to correlate with attention (Fries et al., 2001, 2008). Experiments have implicated gamma oscillations during learning (Bauer et al., 2007) and memory (Pesaran et al., 2002). Numerical studies have demonstrated that coherent gamma oscillations between neuronal populations can provide temporal windows during which information transfer can be enhanced (Womelsdorf et al., 2007). The disruption of gamma frequency synchronization is also concomitant with multiple brain disorders (Bressler, 2003; Baar, 2013; McNally and McCarley, 2016; Krystal et al., 2017; Mably and Colgin, 2018).

Many large-scale network simulations (Traub et al., 2005; Chariker and Young, 2015) and firing rate models (Brunel and Hakim, 1999; Keeley et al., 2019) have been used to capture the wide range of the experimentally observed gamma band activity. The main mechanism underlying the emergent gamma dynamics appears to be the strong coupling between network populations, either via synchronizing inhibition (Whittington et al., 2000) or via competition between excitation and inhibition (Whittington et al., 2000; Chariker and Young, 2015). However, a theoretical account of how the collective behavior emerges from the detailed neuronal properties, local network properties and cortical architecture remains incomplete.

Recently, Young and collaborators have examined the dynamical properties of gamma oscillations in a large-scale neuronal network model of monkey V1 (Chariker et al., 2018). To further theoretical understanding, in Li et al. (2019),introduced a relatively tractable stochastic model of interacting neuronal populations designed to capture the essential network features underlying gamma dynamics. Through numerical simulations and analysis of three dynamical regimes (“homogeneous,” “regular,” and “synchronized”), they identified how conductance properties (essentially, how long after each spike the synaptic interactions are fully felt) can regulate the emergence of gamma frequency synchronization.

Here, we present a sequence of model reductions of the spiking network models based on Li et al. (2019). We first present in detail our methods on a small, homogeneous network of 100 neurons (75 excitatory and 25 inhibitory), exhibiting gamma frequency oscillations. Inspired by Li et al. (2019) and Li and Xu (2019), to achieve dimensional reduction, we assume that the spiking activities during gamma oscillations and their temporally organization are mainly governed by one simple variable, namely, which sub-threshold neurons are only a few postsynaptic spikes from firing. Thus, in terms of dynamical dimensions, the number of network states is drastically reduced (although still too large for any meaningful analytical work, since 2^*n*^ is astronomical even for *n* = 100). Therefore, we further coarse-grain by keeping count of the numbers of neurons that are only a few postsynaptic spikes from threshold. The number of effective states is then reduced to the order of millions. By restricting the dynamics onto this dimensionally-reduced state space, it is now possible to make use of the classical tools of stochastic models to analyze the emergence and statistical properties of gamma frequency synchronization. The reduced models not only successfully capture the key features of gamma oscillations, but strikingly, they also reveal a simple, low-dimensional manifold structure of the emergent dynamics.

The outline of this paper is as follows. In section 2, we present our model reductions, i.e., the sequence of models going from the full model, to the two-state, reduced network model and to its coarse-grained simplification. Especially, we show the low-dimensional manifold representing the gamma dynamics. We discuss the connection of our work to previous studies and how it may benefit future research of gamma dynamics in section 3. Computational methods and technical details are provided in the Methods section.

## 2. Results

In spiking neuronal networks, gamma frequency oscillations appear as temporally repeating, stochastic spiking clusters in the firing patterns. Different mechanisms have been proposed to explain this phenomena. As a minimal mechanism, interneuron network gamma (ING) proposes that the gamma oscillations can be produced by the fast interactions between inhibitory (*I*) neurons alone (Whittington et al., 2000). When inhibitory neurons have intrinsic firing rates higher than the gamma band, they may exhibit gamma firing frequency when mutually inhibited. ING does not require the existence of excitatory (*E*) neurons but studies showed that it loses its coherence in systems where neurons are heterogeneously driven (Wang and Buzsáki, 1996). Another class of theories view the repeating collective spiking clusters as the outcome of competition between *E* neurons and *I* neurons, such as the pyramidal-interneuron network gamma (PING, Whittington et al., 2000; Börgers and Kopell, 2003) and recurrent excitation-inhibition (REI, Chariker et al., 2018).

Though sharing many common qualitative features, PING and REI provide substantially different explanations to the formation of gamma oscillations. Most remarkably, the collective spiking clusters in PING are usually whole-population spikes as a result of steady external input. The *E*-population spikes induce *I*-population spiking activities that are offset in time, and strong enough to suppress the entire network. A new *E*-population spiking event occurs after the inhibition wears off, leading to a series of nearly periodic, whole-population oscillatory activity.

On the other hand, the REI mechanism depicts a highly stochastic network dynamics: Driven by noisy stimulus, a few *E*-neurons crosses the threshold, and the subsequent recurrent excitation recruit more spikes from other excitatory neurons, leading to rapidly rising spike clusters. But this can not go forever since a few of the inhibitory neurons are excited at the same time, pushing the whole population to a less excitable condition. The next collective spiking event then emerges from the victory of excitation during its competition with inhibition. Therefore, the important features of the transient spike clusters are highly temporally variable, and the gamma frequency band of the oscillation is mainly a statistical feature of the emergent complex temporal firing patterns.

Our primary goal is to provide a better understanding of the emergence of gamma oscillations from the interaction between neurons in this non-linear, stochastic, and high-dimensional context. In general, the biggest difficulty of analyzing spiking network dynamics is its dimensionality. Consider a network of *N* neurons, the number of possible states grows exponentially as *N* → ∞, no matter if we use a single-neuron model as complex as the Hodgkin-Huxley (Hodgkin and Huxley, 1952) or as simple as binary-state (Cowan, 1991). Previously, many attempts have been made for model reduction by capturing collective network dynamics in specific dynamical regimes. Successful examples include kinetic theories (Cai et al., 2006) for mean-field firing patterns, statistical field theories (Buice and Chow, 2013) for higher-order correlations, Wilson-Cowan neural field model (Wilson and Cowan, 1972, 1973) for spatial-temporal patterns, to name a few. In this study, our reduced models are developed with similar motivations to capture gamma oscillations. Generally, models incorporating many biologically realistic details can be very complicated; The reduced models are much easier to analyze, but some of the neglected information can lead to biases in many aspects. Here, we aim for a balance between realism and abstraction. Specifically, we present a sequence of reduced models, between which the connections are well defined (and can be mathematically analyzed in future studies). Importantly, we find that even the coarsest model preserves important features and statistics of the gamma oscillations found in the full model.

Our reduced models are based on the integrate-and-fire (IF) model, which is widely employed in previous models of spiking networks (see Methods). In a recent study, gamma oscillations have been found emerging from the simulation of a large-scale IF network model of layer 4Cα of V1 (Chariker et al., 2018). Later theoretical studies suggest that gamma oscillations in different spatial regions decorrelate quickly on the scale of a couple of hypercolumns, echoing experimental observations that gamma oscillation is very local in cortex (Menon et al., 1996; Lee et al., 2003; Goddard et al., 2012). Therefore, we first focus on a small homogeneous network with 75 excitatory neurons (*E*) and 25 inhibitory neurons (*I*) as an analogy of an orientation-preference domain of a hypercolumn in V1.

### 2.1. Reduced Models Captures Statistics of Gamma Oscillations

#### 2.1.1. A Markovian Intergrate-and-Fire Network

We start with the Markovian integrate-and-fire model (MIF) first proposed in Li et al. (2019), and hereafter referred to as the “full model.” As an analogy of the conventional IF model (see Methods), the MIF brings us two additional conveniences: First, the discretized states of Markovian dynamics make theoretical analysis easier as the probability flow from one state to another is now straightforward; Second, the Markov properties of the MIF enable the computation of the invariant measure of gamma oscillations directly from the probability transition matrix.

**Figure 1:**
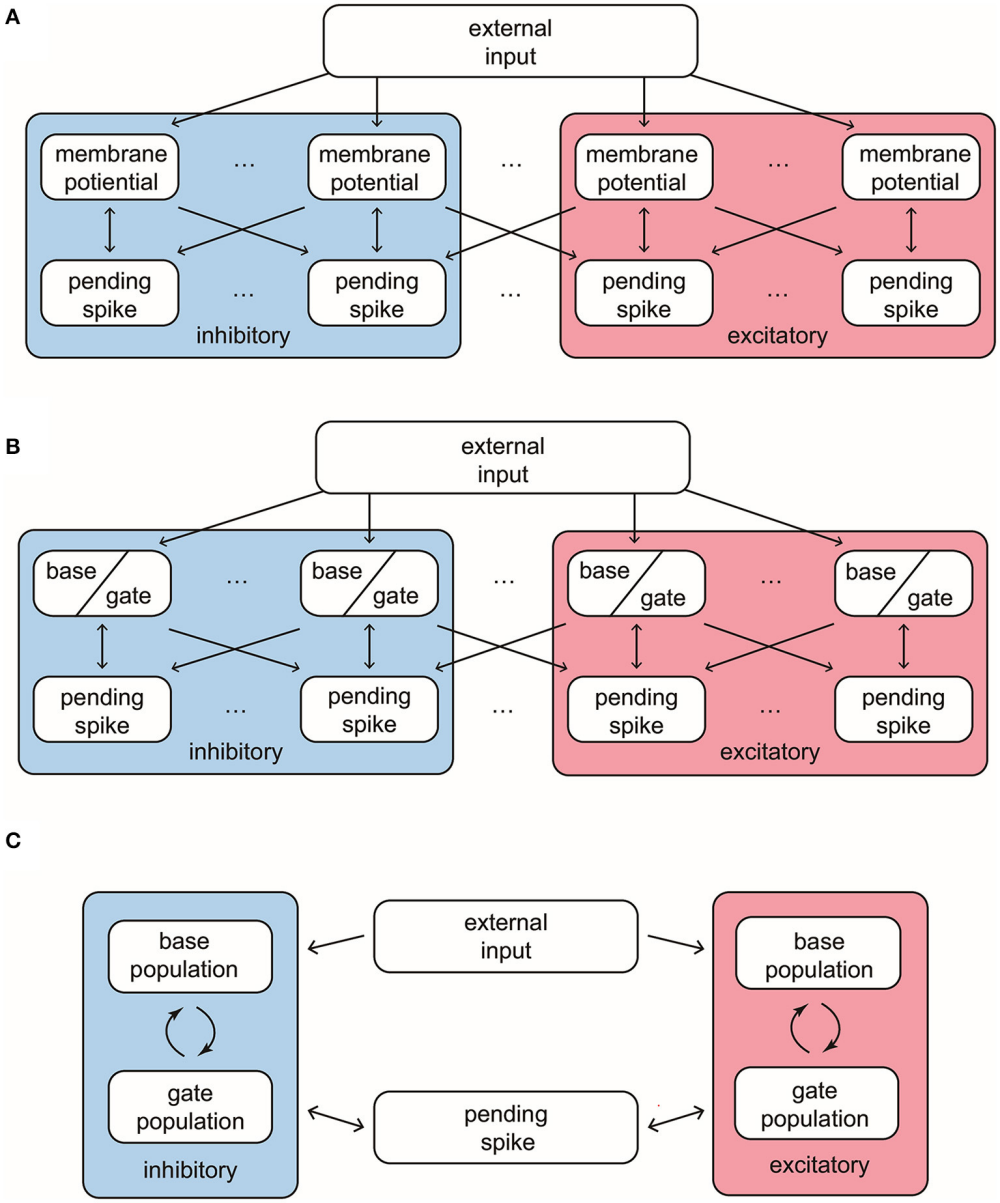
Structures and important features of 3 models. **(A)** The structure of the full Markovian integrate-and-fire network. **(B)** The structure of the reduced network model, where the membrane potentials only take values in {Base, Gate}. **(C)** The structure of coarse grained model. In this model the pending *E*-kick pools for every neuron are merged in to one for the whole network, and so are the pending *I*-kick pools.

Our MIF model network is composed of 100 interacting neurons (75 *E*-neurons and 25 *I*-neurons) driven by external Poissonian stimulus (Figure 1A). The state of neuron *i* is described by three variables $\left(v_{i},H_{i}^{E},H_{i}^{I}\right)$, where *v*_*i*_ represents individual membrane potentials and $H_{i}^{\left\{E,I\right\}}$ are analogies of the *E* and *I* conductances (see below). We use the size of external kicks to discretize the membrane potential, and let *v*_*i*_ range from *V*^*I*^ = −66, the reversal potential of inhibitory synapses, to *V*^*th*^ = 100, the spiking threshold. Immediately after reaching *V*^*th*^, *v*_*i*_ enters the refractory state R, and, at the same time, sends a spike to its postsynaptic targets. After an exponentially distributed waiting-time $\tau^{R}$, *v*_*i*_ resets to the rest potential *V*^*r*^ = 0. The “integrate” part of MIF is separated into two components, external and recurrent. Each external kick increases *v*_*i*_ by 1. To model the effects of recurrent network spikes, we use $H_{i}^{\left\{E,I\right\}}$ to denote the number of postsynaptic spikes (forming “pools” of pending-kicks) received by neuron *i* that has not yet taken effect. When receiving an {*E, I*}-spike, the corresponding pending-kick pool, $H_{i}^{\left\{E,I\right\}}$, increases by 1. Each postsynaptic spike affects *v*_*i*_ independently, and after an exponentially distributed waiting-time (i.e., the synaptic time-scale) τ^{*E,I*}^, increase or decrease *v*_*i*_ (depending on {*E, I*} of the presynaptic neuron). The specific increment/decrement depends on the synaptic strength and the state of *v*_*i*_. The connections between neurons are homogeneous: Whether a spike released by neuron *i* is received by neurons *j* is determined by an independent coin flip, whose probability only depends on the type of neuron *i* and *j*. We leave the details and choices of parameters to Methods.

**Figure 2:**
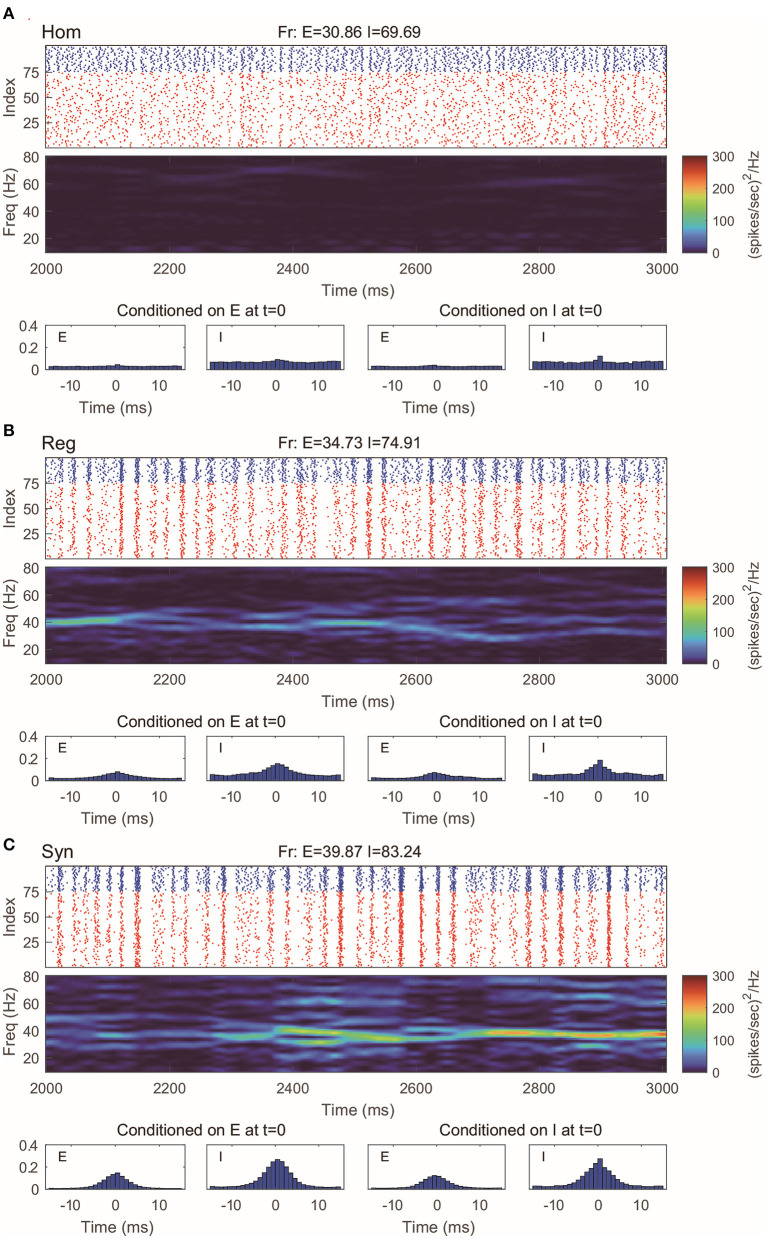
Gamma oscillation exhibited by the MIF network in three different regimes. **(A)** Homogeneous regime (“Hom”). Top: Raster plots of all *E*-neurons (red) and *I*-neurons (blue) with firing rates noted in title. Middle: The spectrogram of the firing pattern exhibiting low density in the gamma band (30–80 Hz). Bottom: The spiking correlation diagrams, which are binned on the distribution of relative spike timing conditioned on E or I spike at *t* = 0. **(B,C)** Same as **(A)**. The regular (“Reg”) and synchronized (“Syn”) regimes exhibit more synchronous firing patterns and stronger gamma-band activity.

This 100-neuron MIF network exhibits gamma-band oscillations as demonstrated in Li et al. (2019) (Figure 2). By varying the synaptic time scales τ^{*E,I*}^, we examine three regimes with different degrees of synchrony: homogeneous (“Hom”), regular (“Reg”), and synchronized (“Syn”). (Specifically, we fix the expectation of the waiting time for *I*-kicks (τ^*I*^) and manipulate separately the expectation of waiting time for *E*-spikes on *E* and *I* neurons, τ^*EE*^ and τ^*IE*^; for full details, see Methods). In the raster plot of “Hom” regime, the MIF network produces a firing pattern in which spikes do not exhibit strong temporal correlations (Figure 2A, top). (This is also verified by the spike-time correlations conditioned on each {*E, I*}-spike; see Figure 2A, bottom). Meanwhile, no strong spectral density peak is seen in the spectrogram (Figure 2A, middle). In the “Reg” regime, however, spikes begin to cluster in time as multiple firing events (hereafter, MFEs) and exhibit stronger spike-time correlations (Rangan and Young, 2013a,b). Namely, MFE is a temporally transient phenomenon lying between homogeneity and total synchrony, where a part (but not all) of the neuronal population fires during a relatively short time window, as widely reported in previous experimental and modeling studies (Beggs and Plenz, 2003; Churchland et al., 2010; Yu and Ferster, 2010; Plenz et al., 2011; Shew et al., 2011; Yu et al., 2011). Affected by MFEs, the mass of the spectral density is primarily located in the gamma band, especially around 40–60 Hz (Figure 2B). Many more and stronger synchronized firing patterns, higher spike-time correlations, and stronger gamma-band spectral peaks are observed in the “Syn” regime. These dynamics and statistics are consistent with the results of Li et al. (2019) and in IF neuronal network simulations (Zhang et al., 2014a,b; Zhang and Rangan, 2015). Furthermore, the gamma-band spectrograms are comparable with experimental studies (Xing et al., 2012).

We note that although our MIF network only consists of *N* = 100 neurons, the number of states in the Markov chain is ${\left(168\cdot n_{H^{E}}\cdot n_{H^{I}}\right)}^{N}$, where $n_{\left\{H^{E},H^{I}\right\}}$ are the largest possible sizes of the pending spike pools (see Methods for precise definition). Therefore, it is computationally unrealistic to do any meaningful analytical work to understand the dynamics, especially how gamma oscillations can emerge from the probability flows between different states. Therefore, we regard this MIF network as the “full model,” and apply dimensional reduction methods for further analysis.

#### 2.1.2. A Reduced Network With Two-State Neurons

First, we introduce a reduced network (RN) consisting of two-state neurons, i.e., RN model has the same setup as the MIF network except that the membrane potentials only have two states: *base* and *gate* (Figure 1B). From the perspective of the full model, a neuron is deemed as a base or gate neuron by how far it is away from firing. Specifically, we set a cutoff *V*^*c*^ below the threshold *V*^*th*^, and neuron *i* is regarded as a gate neuron if $v_{i}>V^{c}$, otherwise it is a base neuron (including $v_{i}\leq V^{c}$ and $v_{i}=R$). Neuron *i* flips between the base and gate states when

1. *v*_*i*_ crosses the cutoff *V*^*c*^, or
2. Neuron *i* fires and enters the refractory state R.

However, the reduction to two-state neurons immediately raises a question: Without the consecutive discrete states between [*V*^*I*^, *V*^*th*^], how can we represent the effect of (external, *E*/*I*-) kicks on individual neurons when *v*_*i*_ only takes two possible states?

Consider an *E*-neuron *i* in the gate state, i.e., $v_{i}>V^{c}$ in the corresponding MIF network. Since we do not know the exact value *a priori*, *v*_*i*_ can take any value between [*V*^*c*^, *V*^*th*^] with a probability determined by the empirical distribution of the whole *E*-population. When an *E*-kick takes effect and increases *v*_*i*_ by a synaptic strength of *S*_*EE*_, neuron *i* fires and changes state to “base” if and only if *v*_*i*_ is located in the excitable region $\left(V^{th}-S_{EE},V^{th}\right]$, otherwise it stays in the gate state (and $v_{i}\in \left[V^{c},V^{th}-S_{EE}\right)$). Therefore, a single *E*-kick has a probability

(1) $$\begin{array}{r} P_{E}^{GE}=\text{P}\left(v_{i}\in \left(V^{th}-S_{EE},V^{th}\right]|v_{i}\in \left(V^{c},V^{th}\right]\right)\\ =\frac{\text{P}\left(v_{i}\in \left(V^{th}-S_{EE},V^{th}\right]\right)}{\text{P}\left(v_{i}\in \left(V^{c},V^{th}\right]\right)}, \end{array}$$

to excite a gate *E*-neuron, leading to its spike and transition to the base state. That is, $P_{E}^{GE}$ is the transition probability of neuron *i* in the excitable region, conditioned on that neuron *i* is a gate neuron. *A priori*, we do not have the full distribution of neuronal states, therefore, in order to close the RN model, we use statistical learning methods by inferring $P_{E}^{GE}$ from a long-term simulation of the full MIF network. Likewise, we acquire all other transition possibilities induced by kicks (see Methods).

This reduction of classifying spiking neurons into two states is based on one simple assumption: The emergence of MFEs in the collective dynamics is mostly sensitive to one variable, i.e., the number of subthreshold neurons are only a few spikes from firing (gate neurons). On the other hand, the distribution of neurons with lower membrane potentials (base neurons) is less immediately relevant since it is much less possible for them to generate spikes in the next few milliseconds. Therefore, the initiation and maintenance of gamma oscillations are dominated by the probability flow between gate and base states.

Here we remark that, at first glance, the need to perform simulations of the full model to learn transition probabilities defeats the main purpose of our model reduction. However, the full-model simulation can be reused when the sub-threshold voltage distributions of different parameter sets share similar geometrical features. For example, in the following model reductions, full-model simulation for the “Syn” regime were exploited to train the RN models for the “Hom” and “Reg” regimes. The reason is that these regimes only differ in the synaptic time scales. The main dynamical interactions, mediated through E/I-kicks, excite/inhibit the neuron populations in similar fashion, thus facilitating our model reductions.

**Figure 3:**
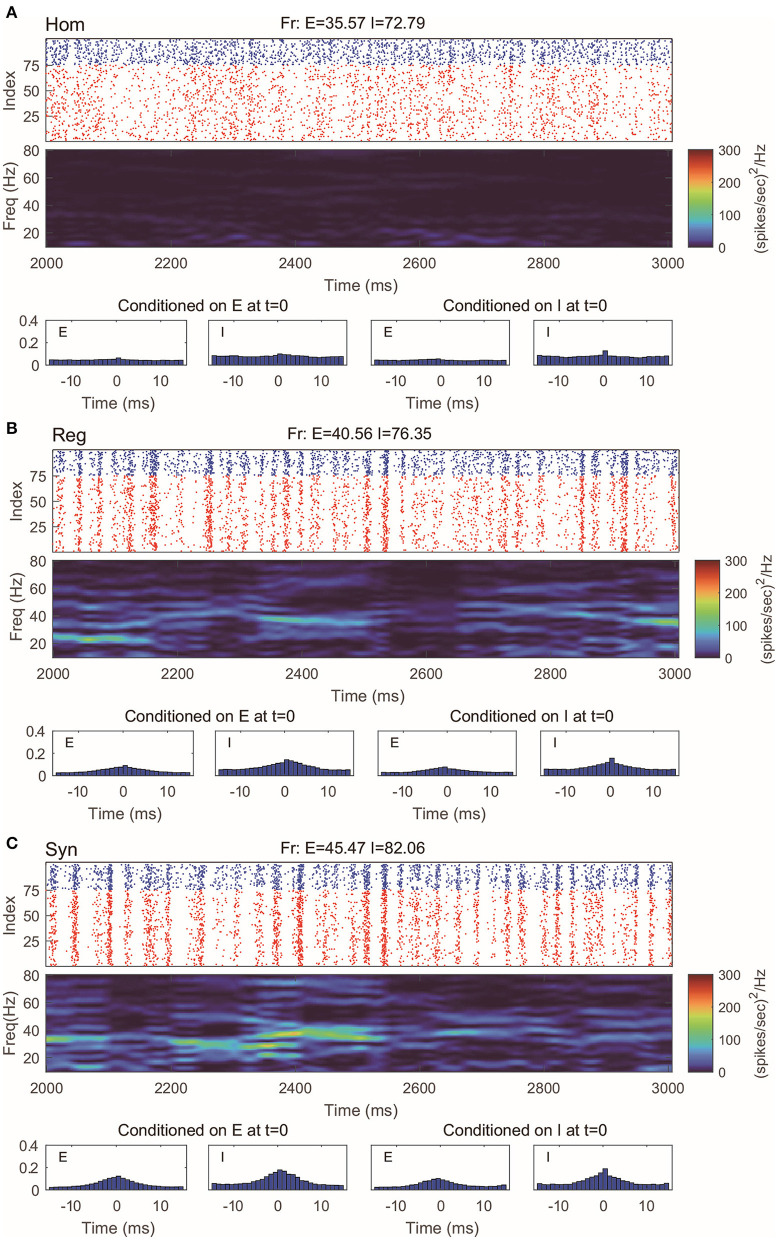
Gamma oscillation captured by the reduced network consisting of two-state neurons. **(A–C)** Same regimes and statistics investigated in Figure 2.

Even with such a drastic simplification, the RN model provides remarkably good approximation of dynamics produced by the MIF network (Figure 3), which is verified by raster plots, firing rates, and spectrum densities similar to those of the full model in Figure 2. On the other hand, we notice that the spike-time correlations in the “Syn” regime are indeed slightly lower than the corresponding value in the full model. This is not very surprising: When the spiking pattern is synchronized, the dynamics is more sensitive to the details of the probability distribution of neurons in the excitable region since one spike may trigger more spikes followed by other neurons. One may, of course, consider using more information to describe the full distribution of the membrane potentials (perhaps by using three or more states instead of two). Though a more detailed models may provide us with better numerical approximations, our primary goal of capturing the key features of gamma oscillation has been well served by the current version of the RN model with two-state neurons.

Though radically simplified, our RN model is still a Markov chain, with ${\left(2\cdot n_{H^{E}}\cdot n_{H^{I}}\right)}^{N}$ states. However, the setup and success of the RN model provide important insights for further model reduction.

#### 2.1.3. A Coarse-Grained Approximation

When we infer the transition probabilities between gate and base states, we are uncertain of the full distribution of *v*_*i*_ in the network (besides the number of neurons in base and gate states). Therefore, the success of the RN model suggests that we may think of the transition probabilities as functions of the number of {*E, I*} neurons in each state: *N*_*GE*_, *N*_*GI*_, *N*_*BE*_, *N*_*BI*_. Thus, the core idea of further simplifying by coarse-grained (CG) approximation is this: Instead of thinking about the state of each individual neuron, we study the state of population statistics. Below we summarize the setup of our CG model and leave the details to Methods.

Let us first consider the pending-kick pools of a single neuron. Take the *I*-to-*E* and *I*-to-*I* projections as an example: Because of the homogeneity of the network, for an *I*-spike, each of its postsynaptic neuron receives it independently with a probability that depends only on the specific neuronal type {*E, I*}. In addition, each spike takes effect independently, with the same waiting time distribution (exponential with mean τ^*I*^). Therefore, this is equivalent to a collective *I*-kick pool of size *H*^*I*^, representing the sum of all *I*-kick pools in the entire network. In this pool, each pending-kick takes effect independently and is randomly distributed to a specific neuron with a probability depending only on its *E*/*I* type. With similar considerations, the *E*-kick pools are also merged into one at a size *H*^*E*^. However, since (τ^*EE*^, τ^*IE*^) are separately manipulated in the full model, we have to ignore the subtle differences between the consumption rates of pending *E*-kicks on *E* and *I* neurons. Specifically, we assume that a constant portion of *H*^*E*^ are distributed to *E* (*I*) neurons (i.e., *H*^*EE*^ = *a*^*EE*^ · *H*^*E*^. See Methods), which introduces a bias in our CG model.

Since each neuron is driven by the same, coarse-grained {*E, I*}-kick pools, due to the interchangeability of *I* neurons, they are now only differentiated by their current state (base or gate). Similarly, the information of the state of every *I* neuron is now represented by the Numbers (*N*_*BI*_, *N*_*GI*_). Since the total number of *I* neurons is fixed, we only need *N*_*GI*_. These considerations also apply to the *E* neurons. Thus, our CG model becomes a Markov chain with four variables:

(2) $$\begin{array}{l} \left(N_{GE},N_{GI},H^{E},H^{I}\right). \end{array}$$

Kicks taking effect may change *N*_*GE*_ and *N*_*GI*_, and spikes released by neurons increase *H*^*E*^ and *H*^*I*^. The CG model contains $N_{E}\cdot N_{I}\cdot N_{H^{E}}\cdot N_{H^{I}}$ states ($N_{H^{E}}=n_{H^{E}}\cdot N$, $N_{H^{I}}=n_{H^{I}}\cdot N$). For each state, there are at most 12 possible transitions to other states (see Methods). Therefore, the CG model becomes an *O*(*N*^4^) problem, allowing us to analyze the dynamics in detail.

**Figure 4:**
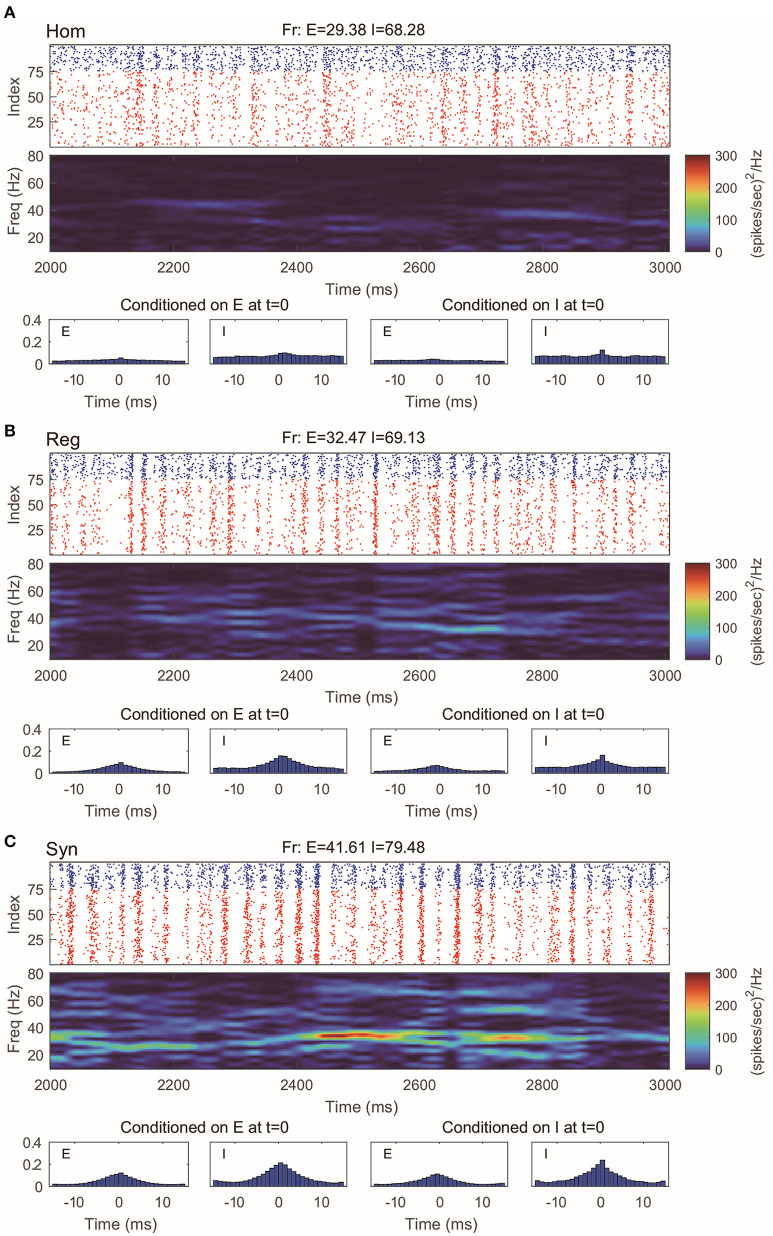
Gamma oscillation captured by the coarse-grained model. **(A–C)** Same regimes and statistics investigated in Figures 1, 2. The fake raster plots are produced by randomly assigning spikes to each neuron.

The CG model is a natural simplification of the RN model, and it is reasonable to expect CG to capture the main features of gamma oscillations. Indeed, we find that in all three regimes we considered, the behaviors of CG model are in well agreement with both the MIF and RN models (Figure 4). Note that, since we do not track the dynamics of individual neurons in CG, the (fake) raster plots are generated by randomly assigning spikes to neurons.

### 2.2. Gamma Dynamical Features in Reduced Models

**Figure 5:**
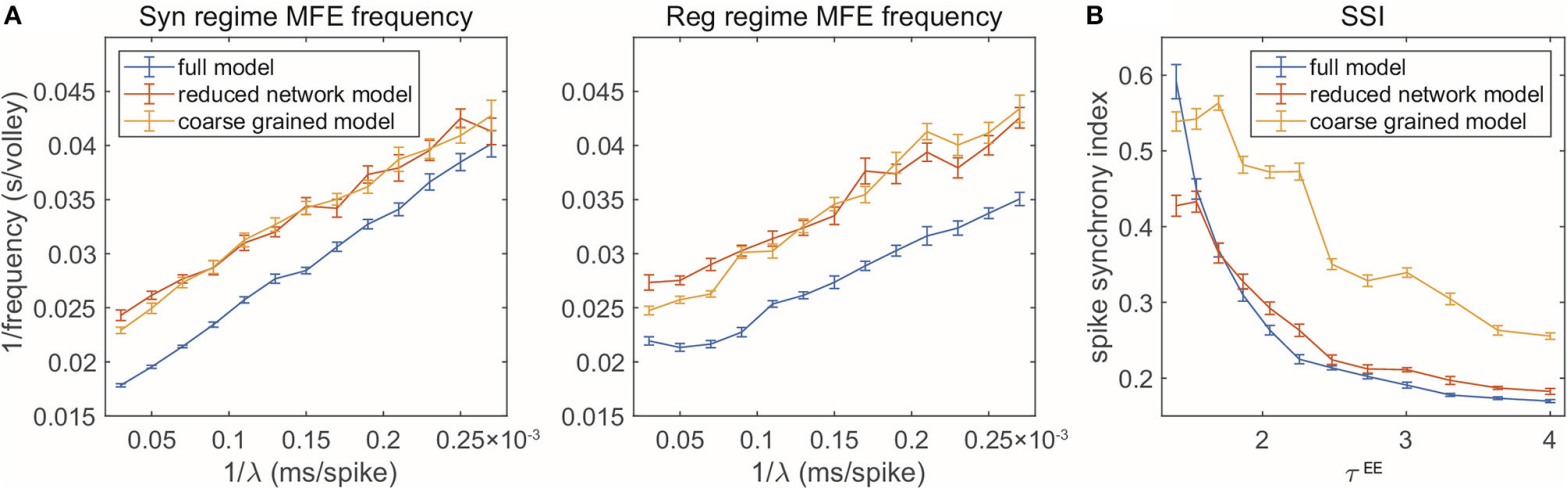
Two gamma features captured by reduced models. **(A)** MFE waiting time linearly related to external stimulation waiting time. Left: Syn regime; Right: Reg regime. **(B)** Degree of synchrony decreases when τ^*EE*^ increases.

The reduced models are not designed to reproduce every detail of the full model. But how well can our model reduction capture the dynamics and key statistical features? In addition to the firing rates, spectral densities, and spike-time correlations for the three selected regimes, we examine the RN and CG models when we change parameters continuously. In this section, we test two dynamical features observed in the full, MIF model. For each parameter set involved, we numerically simulate each of the three models (MIF, RN, and CG), for 10 s each, and divide the dynamics into 10 batches. We then show the batch means and standard errors of the statistics collected from the batches (Figure 5).

First, when the frequency of the external Poisson stimulus (λ) increases, MFEs appear more frequently in the firing patterns. According to Chariker et al. (2018), a new MFE is initiated by excitatory stimulation or by chance when the inhibition from the last MFE fades. Therefore, stronger external stimuli result in faster initiation of a new MFE. From the firing patterns of our three models, we use a spike cluster detection algorithm (Chariker and Young, 2015) to recognize individual MFEs (see Methods) and examine how their emergence is regulated by external stimulus. We find that the RN and CG models capture the same trend exhibited in the full model (Figure 5A), namely, the average waiting time of MFE (1/MFE frequency) is linearly related to the external kicks (λ^−1^). However, while the trend is captured semi-quantitatively, the reduced models exhibit lower MFE frequencies (or higher MFE waiting time in Figure 5A, red and yellow). On the other hand, we find that the MFEs produced by the RN and CG models have longer duration than the MIF model (see Supplementary Materials). These results suggest that, on average, the reduced models have slower probability flows between states.

Second, when the ratio $\frac{\tau^{EE}}{\tau^{I}}$ becomes smaller, the *E*-kicks take effect on *E* neurons relatively faster, and thus, the recurrent network excitation recruits other *E*-spikes on a shorter time scale. Therefore, the whole network exhibits more synchronized firing patterns. This phenomena has been observed in many previous computational models (see, for instance, Keeley et al., 2019), and also verified by the comparison between the three regimes in this paper. Here we go beyond these three regime and change τ^*EE*^ continuously while fixing τ^*I*^ (Figure 5B). In the full model, the degree of synchrony (measured by spike synchrony index, see Methods) exhibits a clear decreasing trend when τ^*EE*^ goes up, which is also well-echoed by the RN model. For the CG model, however, although the same trend is captured, the degree of synchrony is generally higher than in the full model. This bias is introduced when we merge all *E*-kick pools into one and assume $\frac{H^{EE}}{H^{IE}}$ is a constant: The underestimation of *H*^*IE*^ delays the spikes of *I* neurons and the MFEs are artificially prolonged, leading to higher synchrony. To verify this point, we carry out a CG model reduction with five variables $\left(N_{GE},N_{GI},H^{EE},H^{IE},H^{I}\right)$ by keeping separately the pendìng *E*-kick pools of the *E* and *I* populations. This five-variable CG model indeed exhibits much more similar dynamics to full model, including the degree of synchrony (see Supplementary Materials). However, in this study, we choose to focus on the simpler four-variable CG model to facilitate the computation of invariant probability distribution (see section 2.3).

### 2.3. Gamma Oscillations Remain Near a Low-Dimensional Manifold

**Figure 6:**
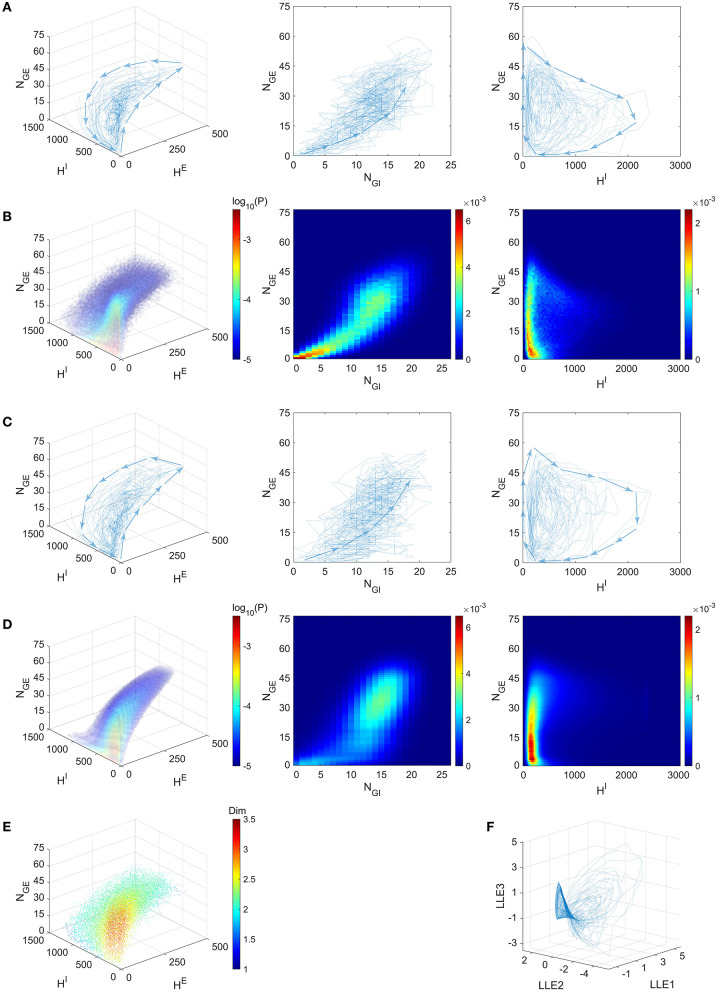
Gamma dynamics restricted on a low-dimensional manifold. **(A)** The trajectories of the full model (Syn regime) presented in the state space of $\left(N_{GE},N_{GI},H^{E},H^{I}\right)$ (blue curves, directions indicated by arrows). Left: The projection on subspace $\left(N_{GE},H^{E},H^{I}\right)$; Middle: The projection on subspace (*N*_*GE*_, *N*_*GI*_); Right: The projection on subspace $\left(N_{GE},H^{I}\right)$. **(B–D)** depict the same subspaces as **(A)**. **(B)** Mass of trajectory density of the full model. **(C)** The trajectories of the CG model (Syn regime). **(D)** The stationary probability distribution of CG model. **(E)** Local dimensionality of data for the full model trajectories, displayed in the same view as **(A)**. **(F)** The full model trajectories are local linearly embedded in a three-dimensional space.

**Figure 7:**
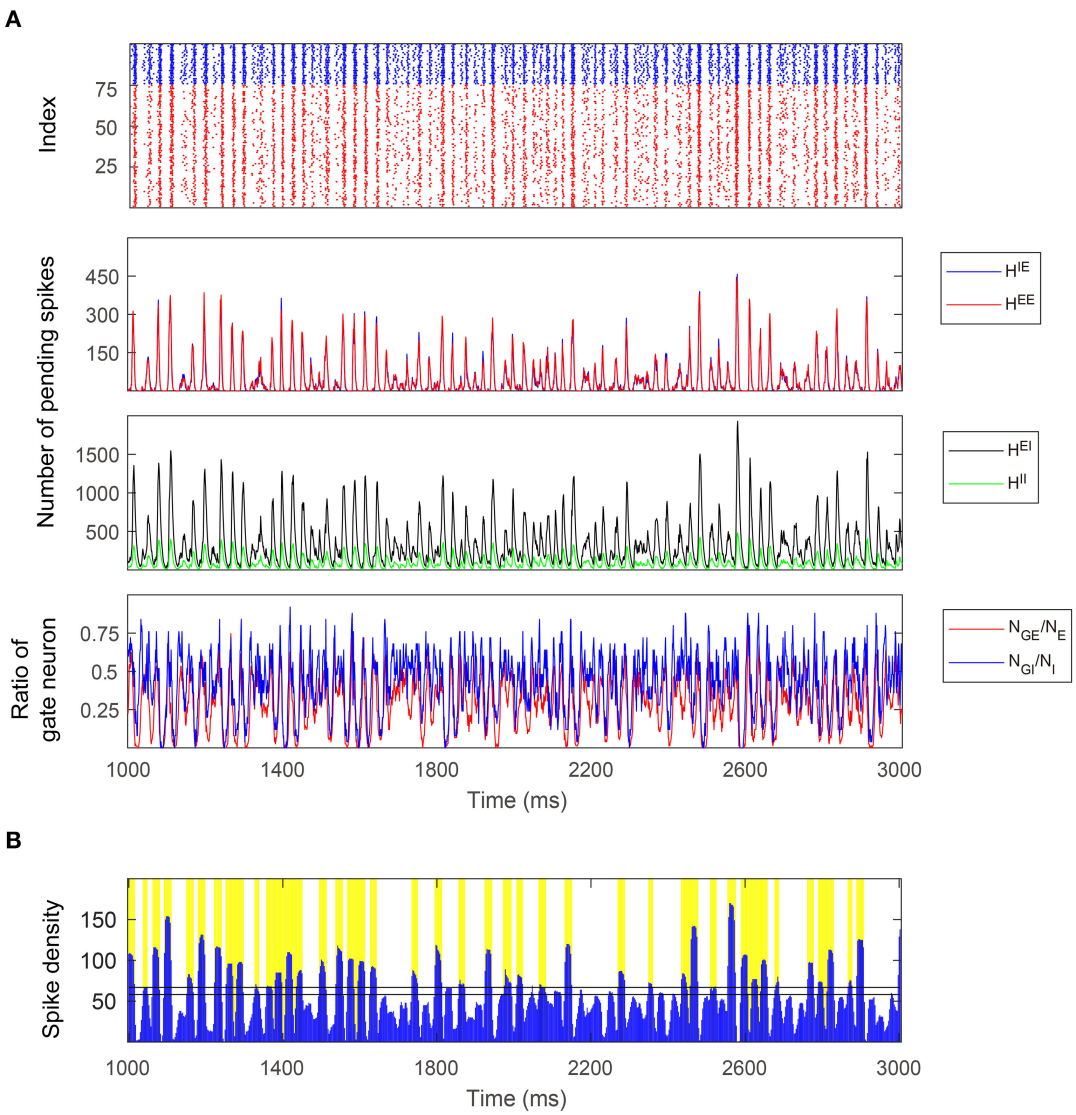
A 2-s simulation of the full model in the Syn regime. **(A)** Top: Raster plot of *E* (red) and *I* (blue) spikes showing gamma-band multiple firing events. Middle: Four types of pending spike pools (*H*^*EE*^, *H*^*IE*^, *H*^*EI*^, *H*^*II*^). The sizes of two *E* pending spike pools are mostly proportional to each other in the Syn regime. Bottom: The proportion of gate neurons in both *E* and *I* populations. **(B)** MFEs are recognized by the spiking volley detection algorithm. Firing events clustering together exhibit high temporal spike densities (blue peaks) and hence labeled as MFEs (yellow bars).

One of our most remarkable finding is that the emergent gamma oscillations from the “Syn” regime in the full model stay near a low-dimensional manifold. Inspired by the fact that the gamma dynamics is successfully captured by the dynamics of only four variables, we simulate the full model in the “Syn” regime, and then project the trajectories onto the 4-dimensional state space suggested by the success of our CG model, i.e., we collect the statistics $\left(N_{GE},N_{GI},H^{E},H^{I}\right)$ from the full model dynamics (Figure 6A; see also Figure 7A).

Since *N*_*GE*_ and *N*_*GI*_ are positively correlated (Figure 6A, Middle; See also Figure 7A, bottom panel), we examine the three-dimensional subspace of $\left(N_{GE},H^{E},H^{I}\right)$. Strikingly, the full model trajectories suggest a low-dimensional dynamical structure of gamma oscillations. This observation is also verified by the mass estimation from the trajectories collected from a long-time (50 s) simulation of the full model (Figure 6B). On the other hand, we also simulate the CG model for 10 s and find similar trajectories in state space (Figure 6C), accounting for its successful reproduction of the emergent gamma dynamics. Since the CG model only consists of *O*(*N*^4^) states, its invariant probability distribution becomes computable from the Markov transition probabilities matrix. Here we present the mass of the distribution after we further “shrink” the CG model and reduce the number of states to millions (Figure 6D). See Methods for the “shrunk” CG model). The invariant probability distribution not only displays similar mass of density as the full model, which the trajectories of the full model remain near (see Supplementary Materials).

The trajectories and the probability distributions of both the full MIF and corresponding CG models reveal a two-dimensional manifold structure with a certain thickness in the orthogonal direction (Figures 6A–D). To verify this point, we carry out a local dimensionality estimation of the full model data (see Methods). Notably, the manifold exhibits a nearly two-dimensional local structure at most of the places (Figure 6E, cyan and green parts), except for a region with low *H*^*E*^, *H*^*I*^ and low-to-medium *N*_*GE*_ (Figure 6E, red part. 0 < *N*_*GE*_ < 30, *H*^*E*^ < 20, *H*^*I*^ < 200). The regions with high dimensionality correspond to the inter-MFE periods (where both number of gate neurons and pending kick pools are small, 0 < *N*_*GE*_ < 15) and the initiation of MFEs (where 15 < *N*_*GE*_ < 30). This is not very surprising: Inter-MFE periods are highly affected by the external stimuli, and the size of each MFE depends on how many *E*-neurons are recurrently recruited by the *E*-spikes at the very beginning. Both processes are highly stochastic, resulting into higher dimensionalities. We note that, this local two-dimensional structure is also verified by the results of local linear embedding (LLE). When we apply LLE to the 10-s full model trajectories (from four-dimensional to three-dimensional), they display a two-dimensional saddle-like manifold as well, in the three-dimensional LLE space (Figure 6F).

The trajectories of the MIF model give us a clear view of the temporal organization of the emergent gamma dynamics. First of all, we observed that (*N*_*GE*_, *N*_*GI*_) are strongly positively correlated (Figure 7A, bottom panel), although *N*_*GI*_ rises slightly faster than *N*_*GE*_ at the initiation of each MFE. In other words, the probability flow of membrane potential distribution of *E* and *I* populations are mostly temporally synchronized, which is also observed in previous computational models (Rangan and Young, 2013a; Zhang and Rangan, 2015; Chariker et al., 2018). In the three-dimensional subspace of $\left(N_{GE},H^{E},H^{I}\right)$, the low-dimensional manifolds help us interpret the gamma dynamics as follows:

1. At the beginning of each MFE, due to the external stimulus and faded recurrent inhibition, the membrane potentials moves up [represented by increasing (*N*_*GE*_, *N*_*GI*_) Figures 6A–D, Middle]. Note that *N*_*GI*_ moves up slightly faster due to the lower *I*-to-*I* connectivity compared to *I*-to-*E*.
2. Some *E* neurons fire, possibly eliciting more spikes from other *E* neurons, so *H*^*E*^ increases fast during this phase.
3. *I* neurons are excited by *H*^*E*^ at the same time, and *H*^*I*^ increases as well. Note that *H*^*E*^ is always consumed faster due to smaller τ^*E*^. In this phase, *H*^*E*^ attains its peak while *H*^*I*^ still increases rapidly.
4. *H*^*E*^ is then consumed and the system is now dominated by the inhibition brought by *H*^*I*^ leading to the end of the MFE. High *H*^*I*^ brings down (*N*_*GE*_, *N*_*GI*_), and terminating the MFE, leading to the inter-MFE-period. The next MFE is unlikely to start until *H*^*I*^ is mostly consumed.

### 2.4. Dimensionality and Entropy

As we have seen above, the full MIF is capable of producing highly heterogeneous dynamical regimes. In particular, Figure 2 illustrated how it is possible to generate gamma oscillations from nearly homogeneous firing with the change of the excitatory time-scale (the expectation of waiting time for *E*-spikes). While some of the features of the emergent gamma oscillations may be attributed to and well-modeled by PING ODE models (with or without noise), the MIF model is capable of generated even more richer network dynamics seen in numerical simulations (see, for instance, Chariker and Young, 2015; Zhang and Rangan, 2015; Chariker et al., 2018; Zhang et al., 2019) and inferred from experimental data (e.g., Xing et al., 2012). Here we would like to demonstrate some of the other possible heterogeneous dynamical regimes, with diverse and wide-ranging dimensionalities. Furthermore, as the MIF explicitly accounts for the effects of randomness in the recurrent network dynamics, therefore, to a large extent, the richness of dynamics can be measured by entropy.

**Figure 8:**
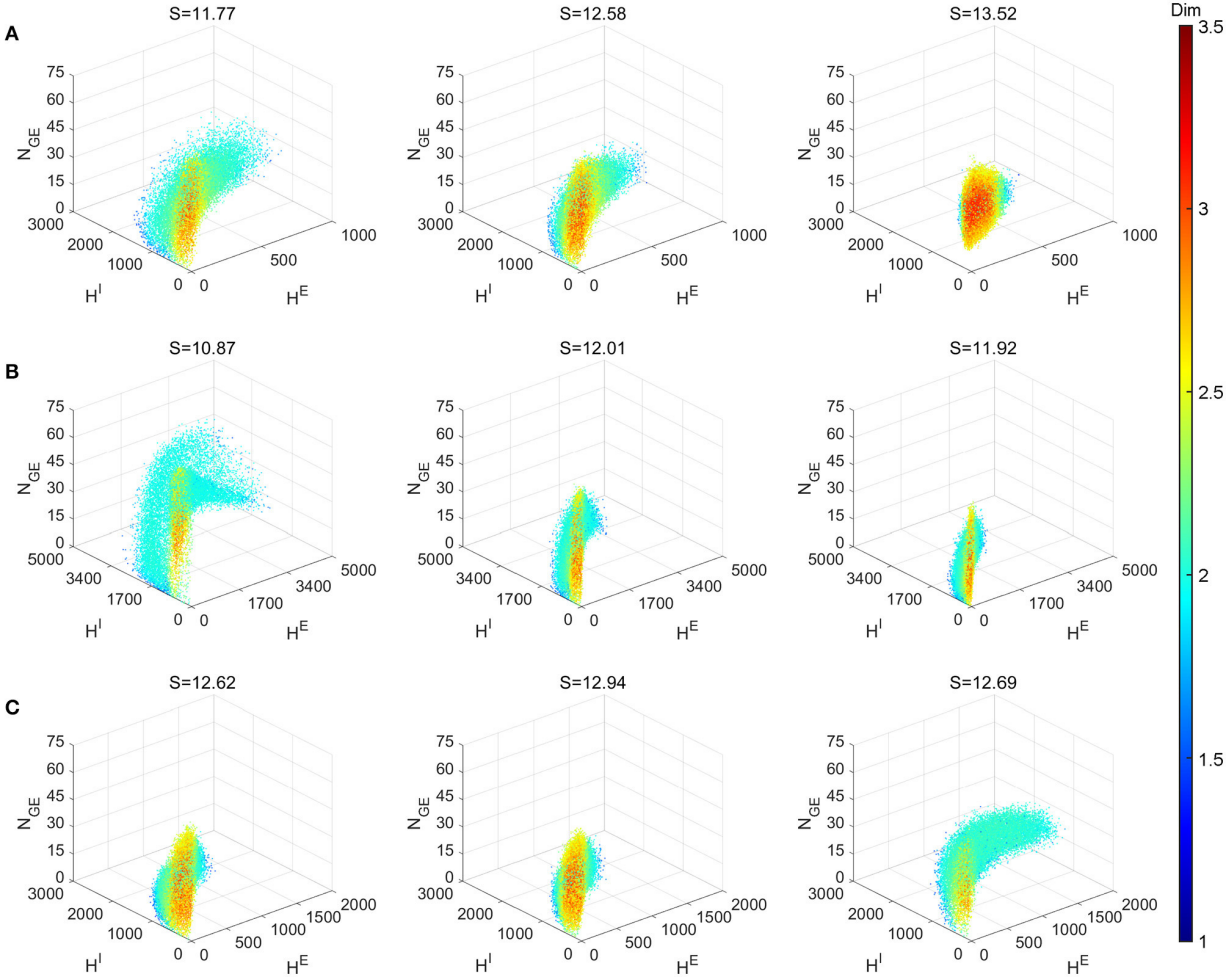
Local dimensionality of more regimes showing the diversity of full model dynamics in different regimes. Entropy *S* of the invariant probability distributions are indicated in the title of each panel. **(A)** Varying τ^*EE*^ parameter. Left to right: Syn, Reg and Hom regimes. **(B)** Setting all connecting probabilities identical. Left to right: 1, 0.5m and 0.25 with *S* × *P* conserved. **(C)**Varying $\tau^{R}$ parameter of E and I neurons. Left to right: $\left(\tau_{E}^{R},\tau_{I}^{R}\right)=\left(2.5,0.5\right)$, (2.5, 2.5), and (0.5, 2.5). Other parameters of **(B,C)** are the same as Syn regime.

Figure 8 explores the various dynamics regimes by changing the excitatory time-scale (Figure 8A), the recurrent synaptic fluctuations (Figure 8B), and the refractory period (Figure 8C). The set of parameter we investigated in (Figures 8B,C) is the same as the Syn regime except for the ones that are varied. In Figure 8A, we project the dynamical trajectories of Figure 2 on the (subspace of the full) state space (namely, the three regimes Syn, Reg, and Hom, from left to right) to characterize and visualize their invariant measures. The change from nearly homogeneous dynamics to stochastic oscillatory gamma is accompanied by an decrease in dimensionality on the invariant measure and its enlargement within state space. This indicates, as one can observe from Figure 2, that the network dynamics is transformed from noisy irregular firing patterns to nearly regular gamma cycles. Not surprisingly, this is also concomitant with an decrease in dynamical entropy. To compare with PING-type ODE models, we simulated the model of Keeley et al. (2019). Even with the addition of stochasticity, the ODE models have dimensions less than 2.5 and entropy less than 9.

Figure 8B demonstrates the effects of the stochasticity in the recurrent synaptic coupling. As we move from left to right, the mean synaptic coupling remains constant (*S*^*QQ*′^ × *P*^*QQ*′^, *Q, Q*′ ∈ {*E, I*}) while the synaptic fluctuations are increased (*P* = 1, 0.5, 0.25). Other parameters remain unchanged from the Syn regime. Therefore, the system with smallest synaptic fluctuation (*P* = 1, left) (Figure 8B left) exhibit the most regular dynamics and much larger gamma cycles than others (indicate by the largest *H*^*E,I*^ numbers). In this case, a small synaptic fluctuation can lead to network synchronization since all neurons receives similar input when one of the presynaptic neuron fires. On the other hand, the case with largest synaptic fluctuation (*P* = 0.25, right) yield smaller and more irregular gamma cycles, accompanied by an increase of dimensionality and entropy of the invariant measures.

Finally, in Figure 8C, we explore the effects of the neuronal refractory periods. As the high-dimensional dynamics emerges from the competition of network excitation and inhibition, the absolute and relative lengths of the refractory periods create a rich assortment of complicated dynamics. Notably, we find that smaller refractory periods for *I* neurons (left, $\tau_{E}^{R}>\tau_{I}^{R}$) incur more regular gamma dynamics (hence, smaller entropy) and larger gamma cycles as well, compared to equal or larger ones (middle, $\tau_{E}^{R}=\tau_{I}^{R}$; right $\tau_{E}^{R}<\tau_{I}^{R}$). To the best of our knowledge, these phenomena have not been discussed by any previous work using ODE models of gamma oscillations, possibly due to the difficulty to reflect these biological details of spiking network models in ODE models with only a few variables.

## 3. Discussion

The vast range of observed neuronal network dynamics presents a tremendous challenge to systems neurophysiologists, data analysts, and computational neuroscientists. Evermore detailed neurophysiological datasets and large-scale neuronal network simulations reveal dynamical interactions on multiple spatial and temporal scales that also participate in essential brain functions. The observed dynamics exhibits rapid, stochastic fluctuations incorporating strong, transient correlations, quite possibly leading to its complexity. Unfortunately, the emergent fluctuating activity cannot effectively be described by standard ensemble averages, as many population methods cannot capture many of the low order statistics associated with the observed dynamical regimes.

The diversity and hierarchy of experimentally observed dynamics in the brain poses this immediate question: What concise, unified mathematical framework can reproduce the co-existence of transient, heterogeneous dynamical states, emerging from a high-dimensional, strongly recurrent neuronal network. Here we focus on gamma frequency oscillations because of their role underlying the transient fluctuations within the stationary states of complex neuronal networks (see, for instance, Buzsáki and Wang, 2012; Siegel et al., 2012), and the belief that they are significant contributors to neural information processing (Fries, 2009; Wang, 2010).

Important theoretical progress has been made by coarse graining sparsely coupled networks (see, for instance, Brunel and Hakim, 1999; Cai et al., 2006, and references therein). These approaches were able to capture spatio-temporal network dynamics dominated by mean-field and uncorrelated synaptic fluctuations. Also influential were the studies focusing on weakly-coupled oscillators. Recent work has made significant theoretical advances by mapping populations of the quadratic IF neurons and the so-called theta neurons to systems of Montbrió et al. (2015) and Laing (2018), where the tools of phase oscillator theory (Ashwin et al., 2016; Bick et al., 2020) can be used to give insight into network synchronization. We view our approach here as complementary to these studies.

Here we demonstrate that, by starting with a Markovian IF model, we can bring the tools of classical Markov processes to bear on stochastic gamma oscillations. Using a model first studied by Li et al. (2019), we show that we can dimensionally reduce and coarse-grain the model network, first, to a two-state reduced network model and then, to a model of transition probabilities between the number of neurons in the two-state, reduced model. The full repertoire of network dynamics between homogeneity and synchrony is faithfully reproduced by our reduced models. Furthermore, by preserving the Markovian dynamics at each step, and combining with data-driven approaches, *we were able to reveal a series of invariant manifolds underlying different type of gamma-band dynamics observed in large-scale numerical simulations. More regular gamma cycles are reflected by the local two-dimensional geometries on the invariant manifolds*.

A tremendous amount of experimental and theoretical literature implicates oscillatory, coherent neural activity as a crucial element of cognition. Here we provided a simple framework through which collective behavior of populations of neurons can be coarse-grained to counting statistics of neurons in specific neuronal states. This dynamical perspective not only can afford a concise handle for the systems neuroscientists, with experimental access to neuronal circuits and populations, but also to the theoreticians, who may wish to build computational and information processing frameworks on top of these coarse-grained, low-dimensional representations.

While we have detailed our methodology for a system with a small number of neurons and homogeneous connectivities, preliminary studies show that we can scale up to much larger networks (see Supplementary Information). We can extend our framework to networks with slowly varying spatial inhomogeneities (e.g., V1 orientation hypercolumns coupled by long-range excitatory connections) and in capturing interneuronal correlations in predominantly feedforward networks, like synfire chains (Diesmann et al., 1999; Wang et al., 2016; Xiao et al., 2017), with each local, nearly homogeneous population described by CG modules (of 2–3 states plus the relevant pending-spike pools). At the same time, we are examining data-driven and machine learning approaches to estimate the various states and transition probabilities directly from numerical simulations, with the hope of applying to neurophysiological data sets. Finally, we are already looking to incorporate higher-order structural motifs in the network connectivity. Higher-order motifs (Song et al., 2005) are likely to substantially influence the types of dynamical correlations within complex networks, and consequently, activate a multitude of spatio-temporal spiking patterns that may have important consequences for information processing and coding in the mammalian brain (Zhao et al., 2011; Hu et al., 2013).

## 4. Methods

### 4.1. Integrate-and-Fire Network

Consider an *N*-neuron Integrate-and-Fire (IF) neuronal network with *N*_*E*_ excitatory neurons (*E*) and *N*_*I*_ inhibitory neurons (*I*), where the membrane potential (*v*_*i*_) of each neuron is driven by a sum of synaptic currents:

(3) $$\begin{array}{l} \;\frac{\text{d}v_{i}}{\text{d}t}=\left(g_{i^{ext}}+g_{i^{E}}\right)\cdot \left(V_{i}^{E-v}\right)+g_{i^{I}}\left(V_{i}^{I-v}\right),\\ g_{i^{ext}}=S_{i^{ext}}\underset{\mu_{i^{ext}}}{\sum }G^{E}\left(t-t_{\mu_{i}^{ext}}\right),\\ \;g_{i^{E}}=\underset{\begin{array}{c} j\in E\\ j\neq i \end{array}}{\sum }S_{{ij}^{E}}\underset{\mu_{j^{E}}}{\sum }G^{E}\left(t-t_{\mu_{j}^{E}}\right),\;g_{i^{I}}=\underset{\begin{array}{c} j\in I\\ j\neq i \end{array}}{\sum }S_{{ij}^{I}}\underset{\mu_{j^{I}}}{\sum }G^{I}\left(t-t_{\mu_{j}^{I}}\right), \end{array}$$

where $g_{i}^{\left\{ext,E,I\right\}}$ are the external, excitatory and inhibitory conductances of neuron *i*. Each neuron receives excitatory spiking stimulus from an external source ($\mu_{i}^{ext}$) and other excitatory/inhibitory neurons in the network $\mu_{j}^{\left\{E,I\right\}}$, where the strength of synaptic couplings are represented by $S_{i}^{ext}$ and $S_{ij}^{\left\{E,I\right\}}$, respectively. A spike is released by neuron *i* when its membrane potential *v*_*i*_ reaches the threshold *V*^*th*^. After this, neuron *i* immediately enters the refractory period, and remains there for a fixed time of $\tau^{R}$ before resetting to rest *V*^*r*^. It is conventional in many previous studies to choose *V*^*th*^ = 1 and *V*^*r*^ = 0 (Cai et al., 2006). Accordingly, *V*^*E*^ = 14/3 and *V*^*I*^ = −2/3 are the excitatory and inhibitory reversal potentials. Each spike changes the postsynaptic conductance with a Green's function,

(4) $$\begin{array}{l} \begin{array}{c} G^{E}\left(t\right)=\frac{1}{\tau^{E}}e^{-t/\tau^{E}}h\left(t\right),\\ G^{I}\left(t\right)=\frac{1}{\tau^{I}}e^{-t/\tau^{I}}h\left(t\right), \end{array} \end{array}$$

where *h*(*t*) is the Heaviside function. The time constants, τ^{*E,I*}^, model the time scale of conductances of the excitatory and inhibitory synapses [such as AMPA and GABA (Gerstner et al., 2014)].

While Equation (3) can model a network with arbitrary connectivity structure, in this paper, we focus on homogeneous networks. That is to say, whether certain spike released by a neuron of type *Q* is received by another neuron of type *Q*′ is only determined by an independent coin flip with a probability $P_{Q^{\prime }Q}$, where *Q, Q*′ ∈ {*E, I*}. Furthermore, $S_{ij}^{\left\{E,I\right\}}$ are also considered as constants independent of *i, j*.

Three different levels of models are illustrated below. The *Markovian integrate-and-fire network* approximates Equation (3) with a Markov process, and the following *reduced network* and *coarse-grained model* are reductions of the full Markovian model.

### 4.2. Full Model: A Markovian Integrate-and-Fire Network

Following a previous study (Li et al., 2019), we rewrite Equation (3) as a Markov process to facilitate theoretical analysis. Therefore, we need to minimize the effects of the memory terms and discretize membrane potentials and conductances. Specifically, *v*_*i*_ takes values in

(5) $$\begin{array}{l} \Gamma :=\left\{V^{I},V^{I}+1,\ldots ,V^{r}-1,V^{r},V^{r}+1,\ldots ,V^{th}\right\}\cup \left\{R\right\}, \end{array}$$

To be consistent with the IF network (3), we choose *V*^*I*^ = −66, *V*^*r*^ = 0, and *V*^*th*^ = 100. As before, *v*_*i*_ enters the refractory state R immediately after reaching *V*^*th*^. However, in this Markovian IF (MIF) network, the total time spent in R is no longer fixed, but an exponential-distributed random variable $\tau^{R}$.

Each neuron receives the external input as an independent Poisson process with rate λ_{*E,I*}_, and *v*_*i*_ goes up by 1 when an external kick arrives. On the other hand, the synaptic conductances of each neuron $g_{i}^{\left\{E,I\right\}}$ are replaced by “pending-kick pools,” $H_{i}^{\left\{E,I\right\}}$. Consider excitatory spikes as an example: Instead of updating $g_{i}^{E}$ with Green's functions when neuron *i* receives an *E*-spike, we add the new spike to an existing spike pool $H_{i}^{E}$. Each spike in the pool will affect *v*_*i*_ independently after an exponentially-distributed waiting time τ^*E*^. $H_{i}^{E}$ is the number of spikes has not taken effect yet. Therefore, for the a sequence of *E*-spikes received by neuron *i*, it is not hard to see that $𝔼\left[H_{i}^{E}\left(t\right)\right]=𝔼\left[g_{i}^{E}\left(t\right)\right]$.

How each spike changes *v*_*i*_ is determined by the type of the spike (*Q*′), the type of neuron *i* (*Q*), and the state of *v*_*i*_. When a spike takes effect, the membrane potential stays unchanged if $v_{i}=R$, otherwise *v*_*i*_ may jump up (for an *E*-spike) or down (for an *I*-spike). On the other hand, the size of each jump depends on the membrane potential, *v*_*i*_, and the synaptic coupling strengths, $S_{QQ^{\prime }}$. For an *E*-spike, *v*_*i*_ increases by *S*_*QE*_. For an *I*-spike, however, the size of the decrement is $\left(v_{i}-V^{I}\right)/\left(V^{th}-V^{I}\right)\cdot S_{QI}$. The difference in the effects of {*E, I*}-spikes is due to the relative values of the reversal potentials. The current induced by $g_{i}^{I}$ is sensitive to *v*_*i*_ while the currents induced by $g_{i}^{E}$ is much less sensitive, i.e., in Equation (3):

(6) $$\begin{array}{l} 0\leq |v_{i}-V^{I}|\leq \frac{5}{3},\;\frac{11}{3}\leq |v_{i}-V^{E}|\leq \frac{16}{3} \end{array}$$

In our MIF network, we take most of the system (synaptic and stimulus) parameters directly from Li et al. (2019), but modified a few to accommodate the smaller network studied here (*N*_*E*_ = 75 vs. *N*_*E*_ = 300 ~ 1000 in Li et al., 2019). The parameters are summarized below:

- Frequencies of external input: λ_*E*_ = λ_*I*_ = 7000 Hz;
- Synaptic strength: *S*_*EE*_ = *S*_*EI*_ = *S*_*II*_ = 20, and *S*_*IE*_ = 8;
- Probability of spike projections: *P*_*EE*_ = 0.15, *P*_*IE*_ = *P*_*EI*_ = 0.5, and *P*_*II*_ = 0.4;
- Synaptic time-scales: τ^*I*^ = 4.5 ms. τ^*E*^ < τ^*I*^ to reflect the fact that AMPA is faster than GABA. We use different choices of τ^*E*^ for regimes implying different dynamics (all indicated in ms):
  1. Homogeneous (“Hom”): τ^*EE*^ = 4, τ^*IE*^ = 1.2,
  2. Regular (“Reg”): τ^*EE*^ = 1.7, τ^*IE*^ = 1.2,
  3. Synchronized (“Syn”): τ^*EE*^ = 1.4, τ^*IE*^ = 1.2.

### 4.3. Reduced Network Consisting of Two-State Neurons

The *reduced network* (RN) is a direct reduction of the MIF network by reducing the size of the state space for membrane potentials. In RN, each neuron *i* is a two-state neuron flipping between “base” or “gate” states, i.e., instead of taking values in the state space Γ, now *v*_*i*_ ∈ Γ_2_ = {*B, G*}. A neuron in the MIF network is deemed “base” or “gate” depending on how likely it is going to fire in the next couple of millisecond: Consider a certain cutoff *V*^*c*^ ∈ Γ, neuron *i* is a gate neuron if $v_{i}\geq V^{c}$ since it is closer to the threshold and is only a couple of *E*-kicks away from spiking. Otherwise, neuron *i* is a base neuron if $v_{i}<V^{c}$ or $v_{i}=R$. Therefore, a flip from base to gate can take place when an *E*-spike or external stimuli takes effect and *v*_*i*_ crosses *V*^*c*^ from the lower side; on the other hand, a flip from gate to base may be due to *v*_*i*_ crossing *V*^*c*^ from the higher side when (1) an *I*-spike takes effect, or (2) the neuron fires and enters the refractory period.

The network with two-state neurons is reduced from the MIF network by combining states together, but generally we do not expect the full MIF model as a lumpable Markov process (Tian and Kannan, 2006). Therefore, the appropriate transition probability between the base and gate states should be carefully estimated so that the RN can correctly capture the dynamics of the MIF network. Since the flip between states can only take place after certain spikes, the possibilities are:

- **Effect of external stimuli**: When a kick arrives, a base {*E, I*} neuron will become a gate {*E, I*} neuron with probability $\left\{P_{ex}^{BE},P_{ex}^{BI}\right\}$, while a gate {*E, I*} neuron will fire and become a base {*E, I*} neuron with probability $\left\{P_{ex}^{GE},P_{ex}^{GI}\right\}$.
- **Effects of E-kicks**: Similar types of transitions here but different probabilities due to different sizes of kicks: $\left\{P_{E}^{GE},P_{E}^{GI},P_{E}^{BI},P_{E}^{BE}\right\}$.
- **Effects of I-kicks**: The I-kicks do not have any effect on a base neuron. But I-kicks will depress a gate neuron to a base neuron with probabilities $\left\{P_{I}^{GE},P_{I}^{GI}\right\}$.

All transition probabilities listed above are time-dependent and determined by the distribution of membrane potentials of neurons in the network. As a first approximation, we can collect their statistics from a long-time simulation of the MIF network. Here we illustrate how to compute $P_{E}^{BE}$ for example, and everything else follows. Consider the distribution of membrane potentials of *E*-neurons, *p*_*E*_(*v*). Then $P_{E}^{BE}$ is the conditional probability a base *E*-neuron goes across *V*^*c*^ within one *E*-kick, which is expressed as:

(7) $$\begin{array}{l} P_{E}^{BE}\left\{p_{E}\right\}=\frac{\int_{V^{c}-S_{EE}}^{V^{c}}p_{E}\left(v\right)\text{ d}v}{\int_{v<V^{c},v=R}p_{E}\left(v\right)\text{ d}v} \end{array}$$

However, in RN, we do not see the exact distribution *p*_*E*_(*v*), but only the number of base and gate *E*-neurons (*N*_*GE*_, *N*_*BE*_) instead. Therefore, to set a closure condition for RN, we consider $P_{E}^{BE}$ as a function of *N*_*BE*_ regardless of the specific distributions, i.e.,

(8) $$\begin{array}{l} \bar{P_{E}^{BE}}(N_{BE})=\text{E}\left[P_{E}^{BE}\left\{p_{E}\right\}|\int_{v<V^{c},v=R}p_{E}(v)\text{ d}v=\frac{N_{BE}}{N_{E}}\right] \end{array}$$

Finally, to collect $\bar{P_{E}^{BE}}\left(N_{BE}\right)$, we run the MIF network simulations for a long time and collect all the events when a *E*-kick takes effect and the membrane potential distribution satisfies the condition listed above. The estimate of $\bar{P_{E}^{BE}}\left(N_{BE}\right)$ is hence the probability of one base *E*-neuron crossing *V*^*c*^ conditioned on these events.

Readers should note that, the three regimes (Hom, Reg, and Syn) investigated in this paper are only differentiated in the waiting time of kicks, i.e., the transition probabilities induced by single kicks are similar, given the observation that the subthreshold distributions in these regimes are alike. Therefore, to carry out reduction in these regimes, we only need the simulation of one canonical parameter set (say Syn) rather than all of them. Similar arguments also apply to different external input rate λ.

### 4.4. A Coarse-Grained Approximation

A *coarse-grained* (CG) approximation is developed to further reduce the number of states of the network. The MIF network has $O\left({\left(168\cdot n_{H}^{2}\right)}^{N}\right)$ states, where *n*_*H*_ is the largest possible number of pending kicks for a neuron. The number for the RN is lower, as $O\left({\left(2\cdot n_{H}^{2}\right)}^{N}\right)$, and yet it still grows exponentially with the size of the network. This number is already astronomical for the 100-neuron network studied in this paper.

A CG approximation of RN model is carried out as follows:

- First of all, due to the homogeneous connectivity of the network, all *I*-neurons have the same probability to add an *E*-kick to their pending-kick pools when an *E*-neuron *i* fires. Since each kick takes effect independently, this is equivalent to a large pool containing all *E*-kicks of the *I*-neurons and each *E*-kicks are randomly distributed to a specific *I*-neuron. Therefore, we only need the size of the large pool *H*^*IE*^ rather than individual *E*-pending-kick pools for each *I* neuron. Likewise, we have pools *H*^*EE*^, *H*^*EI*^, and *H*^*II*^ for other pending spikes. (Note that this CG simplification does not rule out autapses, i.e., the possibility that a spike takes effect on the neuron releasing it. This may be an issue when the network is very small; however, it does not cause any obvious problems in our model with 100 neurons.)
- We now try to further combine the pools. Since all *I*-kicks are consumed with the same waiting time τ^*I*^, we can combine the two *I*-kick pools together. This does not directly apply to the two *E*-kick pools since τ^*EE*^ ≠ τ^*IE*^,i.e., *H*^*EE*^ and *H*^*IE*^ are consumed at different rates. In the CG model, we have to assume the excitatory kicks to *E* and *I* neurons come from the same pool of size *H*^*E*^, and determine *H*^*EE*^ and *H*^*IE*^ by a linear interpolation. Specifically,
  (9) $$\begin{array}{l} H^{EE}=a^{EE}\cdot H^{E},\;H^{IE}=a^{IE}\cdot H^{E}, \end{array}$$
  where the two constants
  (10) $$\begin{array}{l} a^{EE}=\frac{P_{EE}N_{E}}{P_{EE}N_{E}+P_{IE}N_{I}},\;a^{IE}=\frac{P_{IE}N_{I}}{P_{EE}N_{E}+P_{IE}N_{I}} \end{array}$$
  and satisfies *a*^*EE*^ + *a*^*IE*^ = 1. Without doubt, bias of *H*^*EE*^ and *H*^*IE*^ are introduced by this step (larger bias for larger differences between τ^*EE*^ and τ^*IE*^). On the other hand, τ^*EE*^ and τ^*IE*^ are closer in the Syn regime (compared to Hom and Reg, see parameters in section 4.2), i.e., *H*^*EE*^/*H*^*IE*^ ≈ const. Therefore, the introduced bias is smaller in this regime (Figure 7A. See also Figure 5B). In all,
  (11) $$\begin{array}{l} H^{E}=H^{EE}+H^{IE}=\overset{N}{\underset{i=1}{\sum }}H_{i}^{E},\;H^{I}=H^{EI}+H^{II}=\overset{N}{\underset{i=1}{\sum }}H_{i}^{I}, \end{array}$$
  where *H*^{*E,I*}^ are the sizes of the {*E, I*}-kick pools for the whole network.
- Finally, by definition, the transition probabilities of the two-state neurons are functions of the number of gate neurons (see Equation 8). Therefore, instead of the states of each individual neuron, the distributions of membrane potentials can be determined by the numbers of gate neurons (*N*_*GE*_, *N*_*GI*_). Once they are computed, the number of base neurons is given by
  (12) $$\begin{array}{l} N_{GE}+N_{BE}=N_{E},\;N_{GI}+N_{BI}=N_{I} \end{array}$$

Therefore, the CG approximation above is a Markov process with only four variables, two for the number of gate neurons and two for pending kicks $\left(N_{GE},N_{GI},H^{E},H^{I}\right)$, and the number of states is $O\left(N^{4}\right)=N_{E}\cdot N_{I}\cdot {\left(n_{H}\right)}^{2}$. This is a tremendous reduction from exponential to polynomial scaling in the size of the network. We here provide a qualitative description of the dynamics of the coarse-grained model: When an {*E, I*}-kick takes effect, the number of *H*^{*E,I*}^ decreases by one, and the target neuron flips between base/gate states with the probability given by (*N*_*GE*_, *N*_*GI*_) (see **Table 2**); If an {*E, I*}-neuron fires, a spike is released and the pending-kick pool *H*^{*E,I*}^ expands by

(13) $$\begin{array}{l} M_{E}=P_{EE}N_{E}+P_{IE}N_{I},\;M_{I}=P_{EI}N_{E}+P_{II}N_{I}, \end{array}$$

where *M*_{*E,I*}_ is the average number of postsynaptic neuron recipients of an {*E, I*}-spike.

We list all possible transitions from state $X=\left(N_{GE},N_{GI},H^{E},H^{I}\right)$ (Table 1). In addition, since all state transitions are triggered by certain kicks, which take effect independently with exponential waiting time, it is more important to know the transition rates based on the transition probabilities. We list these rates in Table 2.

**Table 1:** All possible transitions from state $X=\left(N_{GE},N_{GI},H^{E},H^{I}\right)$ to another.

**External kick takes effect**	**One *E*-kick takes effect**	**One *I*-kick takes effect**
*N*_*GE*_ + 1	$N_{GE}+1\;H^{E}-1$	$N_{GE}-1\;H^{I}-1$
*N*_*GI*_ + 1	$N_{GI}+1\;H^{E}-1$	$N_{GI}-1\;H^{I}-1$
$N_{GE}-1\;H^{E}+M_{E}$	$N_{GE}-1\;H^{E}-1+M_{E}$	*H*^*I*^ − 1
$N_{GI}-1\;H^{I}+M_{I}$	$N_{GI}-1\;H^{E}-1\;H^{I}+M_{I}$	
Remain	*H*^*E*^ − 1	

*There are 13 cases in all. Column 1: When an external kick takes effect, X may jump to five possible states due to the following reasons: an E base neuron to gate; an I base neuron to gate; an E gate neuron fires and release M_E_ pending spikes to the E pool; an I gate neuron fires and release M_I_ pending spikes to the I pool; or simply stay at the same state. Column 2: Similar as column 1, but all the state transitions are accompanied by the consumption of one E-kick, H^E^ decreases by 1. Column 3: Since a base neuron stays “base” when an I kick takes effect, there are only three possible state transitions in this case*.

**Table 2:** The transition rates of all transitions in Table 1.

**External kick takes effect**	**One *E*-kick takes effect**	**One *I*-kick takes effect**
$P_{ex}^{BE}N_{BE}\cdot \lambda_{E}$	$P_{E}^{BE}a_{EE}\frac{N_{BE}}{N_{E}}H^{E}/\tau^{EE}$	$P_{I}^{GE}a_{EI}\frac{N_{GE}}{N_{E}}H^{I}/\tau^{I}$
$P_{ex}^{BI}N_{BI}\cdot \lambda_{I}$	$P_{E}^{BI}a_{IE}\frac{N_{BI}}{N_{I}}H^{E}/\tau^{IE}$	$P_{I}^{GI}a_{II}\frac{N_{GI}}{N_{I}}H^{I}/\tau^{I}$
$P_{ex}^{GE}N_{GE}\cdot \lambda_{E}$	$P_{E}^{GE}a_{EE}\frac{N_{GE}}{N_{E}}H^{E}/\tau^{EE}$	$\left(1-P_{I}^{GE}a_{EI}\frac{N_{GE}}{N_{E}}-P_{I}^{GI}a_{II}\frac{N_{GI}}{N_{I}}\right)\cdot H^{I}/\tau^{I}$
$P_{ex}^{GI}N_{GI}\cdot \lambda_{I}$	$P_{E}^{GI}a_{IE}\frac{N_{GI}}{N_{I}}H^{E}/\tau^{IE}$	
$\begin{array}{ccc} & \left(1-P_{ex}^{BE}\right)N_{BE}\cdot \lambda_{E} & \\ + & \left(1-P_{ex}^{BI}\right)N_{BI}\cdot \lambda_{I}\\ + & \left(1-P_{ex}^{GE}\right)N_{GE}\cdot \lambda_{E}\\ + & \left(1-P_{ex}^{GI}\right)N_{GI}\cdot \lambda_{I} \end{array}$	$\begin{array}{ccc} & \left(1-P_{E}^{BE}\right)a_{EE}\frac{N_{BE}}{N_{E}}H^{E}/\tau^{EE} & \\ + & \left(1-P_{E}^{BI}\right)a_{IE}\frac{N_{BI}}{N_{I}}H^{E}/\tau^{IE}\\ + & \left(1-P_{E}^{GE}\right)a_{EE}\frac{N_{GE}}{N_{E}}H^{E}/\tau^{EE}\\ + & \left(1-P_{E}^{GI}\right)a_{IE}\frac{N_{GI}}{N_{I}}H^{E}/\tau^{IE} \end{array}$	

### 4.5. Statistics

To quantify how well the reduced models (RN and CG) capture the dynamical features of the full model (MIF), we compare several statistics of the network dynamics (or more precisely, the spiking pattern produced by the network) collected from the simulations of the MIF, RN, and CG models. The reader should note that we can not tell the specific neuron indices of firing events in the CG model; yet it does not affect the computation of the statistics below. The raster plots produced for the CG model, however, are indeed mock-up raster plots, drawn by assigning spikes to neurons randomly among the appropriate {*E, I*} population.

#### 4.5.1. Firing Rates

Spikes from {*E, I*}-cells are collected separately, and firing rates (*fr*_*E*_, *fr*_*I*_) are computed as the average numbers of spikes per neuron per second. All three models are simulated for over 3 s and spikes are collected from the 2nd second to rule out possible influences by the choice of initial conditions.

#### 4.5.2. Spike Synchrony Index

We borrow the definition of spike synchrony index (SSI) from Chariker et al. (2018). SSI describes the degree of synchrony of the firing events as the following. For each spike occurred at *t*, consider a *w*-ms time window centered by the spike (*t* − *w*/2, *t* + *w*/2) and count the fraction of neurons in the whole network firing in such window. Finally, the SSI is the fraction averaged over all spikes.

It is not hard to see that SSI is larger for more synchronous spiking patterns. For the completely synchronized dynamics, every other neuron fires within the time window of each spike hence SSI=1. For completely uncorrelated firing patterns such as Poisson, SSI is a small number close to 0. One should note that the absolute value of SSI depended on the choice of the window size, and we choose *w* = 5 ms (the same as Chariker et al., 2018).

#### 4.5.3. Spectrogram

The power spectrum density (PSD) measures the variance in a signal as a function of frequency. In this study, the PSD is computed as follows:

A time interval (0, *T*) is divide into time bins *B*_*n*_ = [(*n* − 1)Δ*t, n*Δ*t*], *n* = 1, 2, ..., the spike density μ_*n*_ per neuron in *B*_*n*_ is given by μ_*n*_ = *m*_*n*_/*N*Δ*t* where *m*_*n*_ is the total number of spikes fired in bin *B*_*n*_. Hence, the discrete Fourier transform of {μ_*n*_} on (0, *T*) is given as:

(14) $$\begin{array}{l} \hat{\mu }\left(k\right)=\frac{1}{\sqrt{T}}\overset{T/\Delta t}{\underset{n=1}{\sum }}\mu_{n}\Delta te^{-k\cdot \left(2\pi i\right)\cdot \left(n\Delta t\right)}. \end{array}$$

Finally, as a function of *k*, PSD is the “power” concentrated at frequency *k*, i.e., $|\hat{\mu }\left(k\right){|}^{2}$.

#### 4.5.4. Spike Timing Correlations

The correlation diagrams describe the averaged correlation between each spike and others. Consider the correlation with *I*-spikes conditioned on E at t = 0:

For each *E*-spike at time *t*, we take *I*-spikes within the time window [*t* − 15ms, *t* + 15ms], and compute the fraction of *I*-spikes in each 1-ms time bin. The correlation diagrams is then averaged over all *E*-spikes in this simulation.

#### 4.5.5. Spiking Volley Detection

The method defining spiking volleys (or MFEs) is borrowed from Chariker and Young (2015). The core idea of this method is to find time intervals with some length constraints that the firing rate of each time bin in this interval is a certain amount higher than the average firing rate. This is then defined as a spiking volley. We choose 1 ms time bin and δ = 0.33, ϵ = 8 as parameters (Figure 7B).

#### 4.5.6. Dimensionality of Data

We use local principal component analysis (local PCA) method to compute the local dimensionality of the data at each data point *x*, i.e., how its neighbors cluster around *x*. The data points processed here is selected from original data points with probability proportional to the square of distance from the place with highest density mass, in order to make the distribution of data points more uniform which can result in more precise local dimensionality characterization. For *x*, consider the correlation matrix *C*_*x*_ of *x* and its *K* nearest neighbors (*K* selected as 100). Then the dimensionality at *x* is computed as

(15) $$\begin{array}{l} \text{Dim}\left(x\right)=\frac{\text{Tr}{\left(C_{x}\right)}^{2}}{\text{Tr}\left(C_{x}^{2}\right)}=\frac{{\left(\sum_{i}\lambda_{i}\right)}^{2}}{\sum_{i}\lambda_{i}^{2}} \end{array}$$

where λ_*i*_ is the *i*-th eigenvalue of correlation matrix *C*_*x*_. Equation (15) is widely used as the dimensionality definition in theoretical and experimental neuroscience studies (Mazzucato et al., 2016; Gao et al., 2017; Litwin-Kumar et al., 2017; Recanatesi et al., 2019).

#### 4.5.7. Entropy of trajectories

Note that estimating the entropy analytically for a dynamical system can be hard, in this paper, we focus on estimating entropy based on trajectories from 10-s simulations for each regime investigated in Figure 8. To make the entropy computation of our full model and the ODE model for PING comparable, we first extract the four dimension trajectories of all models (for ODE model, we choose the four quantities comparable to ours). Then the space is confined to a 4-D cube with the range of each dimension is the minimum and maximum of the corresponding component of the trajectory data. The space is further uniformly divided into an *n*^4^ (we choose *n* to be the same as *N*_*I*_) grid and we calculate the distribution of the data in that grid, then naturally get entropy as

$$S=\underset{\left(i,j,k,l\right)}{\sum }-P_{i,j,k,l}\log_{2}P_{i,j,k,l}$$

### 4.6. Computational Methods

#### 4.6.1. Exact Timing of Events

During our simulation, we can compute the exact timing of all events including firing, kicks taking effect, etc. We note that MIF, RN, and CG models are all Markov processes whose randomness is mainly due to the exponential distributions of various waiting times. Consider two independent events *A* and *B* with waiting time *X*_*A*_ ~ exp(λ_*A*_), *X*_*B*_ ~ exp(λ_*B*_), we have min{*X*_*A*_, *X*_*B*_} ~ exp(λ_*A*_ + λ_*B*_), i.e., the waiting time for either the first event is also an exponential distribution. Furthermore, the probability for the first occurring events to be *A* is λ_*A*_/(λ_*A*_ + λ_*B*_).

Similar arguments extend to *m* events. By noticing that exponential distributed waiting times are temporally memoryless, we can simulated all three models by repeatedly selecting the first occurring event and generate the actual waiting time by sampling from certain exponential distributions.

#### 4.6.2. Invariant Probability Distributions

After determining the total number of states and the transition probabilities between them, we can calculate the invariant probability distribution from the CG model by computing the eigenvectors and eigenvalues of the transition probability matrix. Such methods can be found in standard linear algebra textbooks such as Roman et al. (2005).

Readers should note that theoretically, there is no upper bound for {*H*^*E*^, *H*^*I*^}. In order to close the computation of invariant probability distributions, we set the $n_{\left\{H^{E},H^{I}\right\}}$, the largest numbers of pending spikes shown up in the simulations as the “boundaries” of the state space. Specifically, the transition probability from state *X* to *Y* is 0 if

1. $X=\left(N_{GE},N_{GI},n_{H^{E}},H^{I}\right)$ and $Y=\left(N_{GE},N_{GI},n_{H^{E}}+a,H^{I}\right)$, or
2. $X=\left(N_{GE},N_{GI},H^{E},n_{H^{I}}\right)$ and $Y=\left(N_{GE},N_{GI},H^{E},n_{H^{I}}+b\right)$,

where *a, b* > 0.

#### 4.6.3. A “Shrunk” Coarse Grained Model

After the reductions, the CG model studied in this paper still has *M* = 5.6 × 10^9^ states. Though the first left eigenvector (i.e., the stationary probability distribution, corresponding to eigenvalue 1) of a sparse, *M*-by-*M* probability transition matrix is computable, the cost could be high for a desktop. Therefore, we aim at an even coarser version of the CG model: a “shrunk” coarse grained (SCG) model. Since *H*^{*E,I*}^ are much higher than *N*_{*GE,GI*}_, we shrink the CG model by combining every *K* states of pending kicks into one, i.e., all $1\leq H_{\text{cg}}^{E}\leq K$ states in CG model is considered as $H_{\text{scg}}^{E}=1$ in SCG model. The intuition is the following: no need to characterize the states of pending spikes very precisely especially when the number is very large (i.e., the difference between 3000 and 3001 is very small). Every state of SCG model can also be represented as a quadruplet ${\mathbb{Q}}_{\text{scg}}=\left(N_{GE},N_{GI},H_{\text{scg}}^{E},H_{\text{scg}}^{I}\right)$. The SCG model works as follow. Firstly the quadruple ℚ_scg_ is lifted to another quadruplet ${\mathbb{Q}}_{\text{cg}}=\left(N_{GE},N_{GI},H_{\text{cg}}^{E},H_{\text{cg}}^{I}\right)$, where $H_{\text{cg}}^{E,I}=\left(H_{\text{scg}}^{E,I}-0.5\right)\cdot K$. Then quadruplet ℚ_cg_ acts following the same rule as the CG model. Lastly the change of ℚ_cg_ is projected back into the change of ℚ_scg_:

1. The change of *N*_*GE*_ and *N*_*GI*_ in ℚ_cg_ is kept the same on corresponding elements in ℚ_scg_;
2. The change *x* of $H_{\text{cg}}^{E,I}$ is replaced by change *y* = [*x*/*K*] + *b* on $H_{\text{scg}}^{E,I}$, where [·] denotes the least integer function and *b* is a Bernoulli variable with probability *p* = (*x*/*K*) − *y*;

Through this model, we can further reduce the *M* states of CG model to *M*/*K*^2^ states of SCG model.

#### 4.6.4. Locally-Linear Embedding

LLE is a non-linear dimension reduction method which discovers the low-dimensional structure of high-dimensional data (Roweis and Saul, 2000). More precisely, it maps the high-dimensional input data into a low-dimensional space. The core idea of LLE is to maintain the local linear structure through the mapping and this is achieved by a two-step optimization. There are *N* input data and we denote the high-dimensional input as ${\vec{X}}_{i}$ and low-dimensional output as ${\vec{Y}}_{i}$. The algorithm is described below and more details see (Roweis and Saul, 2000; Saul and Roweis, 2000).

1. Find the nearest *k* neighbors $S_{i}^{k}$ of each data point ${\vec{X}}_{i}$;
2. $W={\arg\min}_{W^{\prime }}\sum_{i}||{\vec{X}}_{i}-\sum_{j}W_{ij}^{\prime }{\vec{X}}_{j}|{|}^{2}$,$\;W_{ij}^{\prime }=0$ if ${\vec{X}}_{j}\notin S_{i}^{k}$;
3. $Y={\arg\min}_{Y^{\prime }}\sum_{i}||{\vec{Y}}_{i}^{\prime }-\sum_{j}W_{ij}{\vec{Y}}_{j}^{\prime }|{|}^{2}$, subject to $\sum_{i}{\vec{Y}}_{i}^{\prime }=0$ and $\frac{1}{N}\sum_{i}{\vec{Y}}_{i}^{\prime }{\vec{Y}}_{i}^{\prime T}=I$.

## Data Availability Statement

The datasets presented in this study can be found in online repositories. The names of the repository/repositories and accession number(s) can be found at: https://github.com/faro1219/Neural-Oscillations.git.

## Author Contributions

Z-CX conceived the original ideas and developed the theoretical formalism encouraged by and with help of LT. YC and TW wrote code, developed algorithms, and performed numerical computations. LT and Z-CX wrote the manuscript with input from all authors. This project was supervised by LT and Z-CX.

## Conflict of Interest

The authors declare that the research was conducted in the absence of any commercial or financial relationships that could be construed as a potential conflict of interest.

## Publisher's Note

All claims expressed in this article are solely those of the authors and do not necessarily represent those of their affiliated organizations, or those of the publisher, the editors and the reviewers. Any product that may be evaluated in this article, or claim that may be made by its manufacturer, is not guaranteed or endorsed by the publisher.
