# Supplementary material for: Model Reduction Captures Stochastic Gamma Oscillations on Low-Dimensional Manifolds
Source: Front Comput Neurosci. 2021 Aug 17;15:678688. doi: 10.3389/fncom.2021.678688 (PMC8418102; doi:10.3389/fncom.2021.678688)
Supplement: Supplementary file 1 [file Data_Sheet_1.PDF]

frontiers

Supplementary Material

1 LOW-DIMENSION MANIFOLDS

Trajectories of MIF and CG models. Although MIF and CG models exhibit subtle difference in the statistics of the dynamics, we find that their trajectories in state space $(N_{GE}, N_{GI}, H^E, H^I)$ stay close to similar manifolds (Fig. S1).

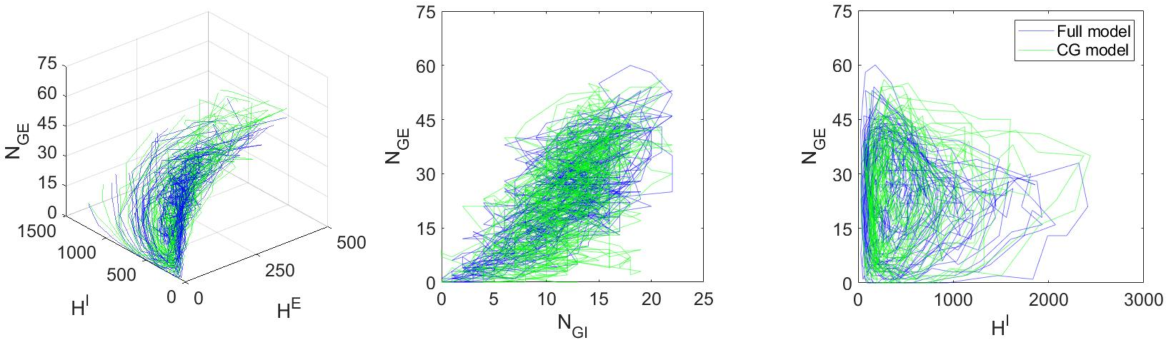

Figure S1. MIF and CG models produce trajectories on similar surfaces. **Left:** The projection on subspace (N_{GE}, H^E, H^I); **Middle:** The projection on subspace (N_{GE}, N_{GI}); **Right:** The projection on subspace (N_{GE}, H^I).

MIF trajectories in Syn, Reg, and Hom regimes. The low-dimensional manifolds in state spaces are powerful tools to visualize and analyze gamma dynamics. Here, we are interested in the comparison of manifolds for these three regimes studied in the manuscript. In the Syn regime, X has a faster speed to consume the pending E-kicks (compared to Hom and Reg), i.e., E-neurons are faster recruited by the recurrent excitation, resulting in larger MFEs. These features are reflected by the full-model trajectories in the three different regimes (Fig. S2).

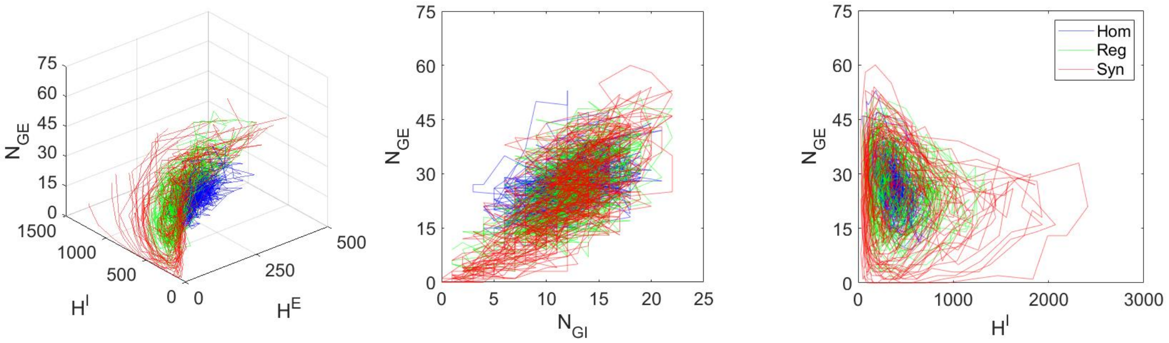

Figure S2. Full model trajectories of three regimes (Syn, Reg, and Hom). **Left:** The projection on subspace (N_{GE}, H^E, H^I); **Middle:** The projection on subspace (N_{GE}, N_{GI}); **Right:** The projection on subspace (N_{GE}, H^I).

Convergence of MIF and CG models. In Fig. S3, we use trajectories in different time intervals to demonstrate the convergence rate of the full and CG models (in Syn regime). The similarities between the distributions of trajectories indicate quick convergence of both models.

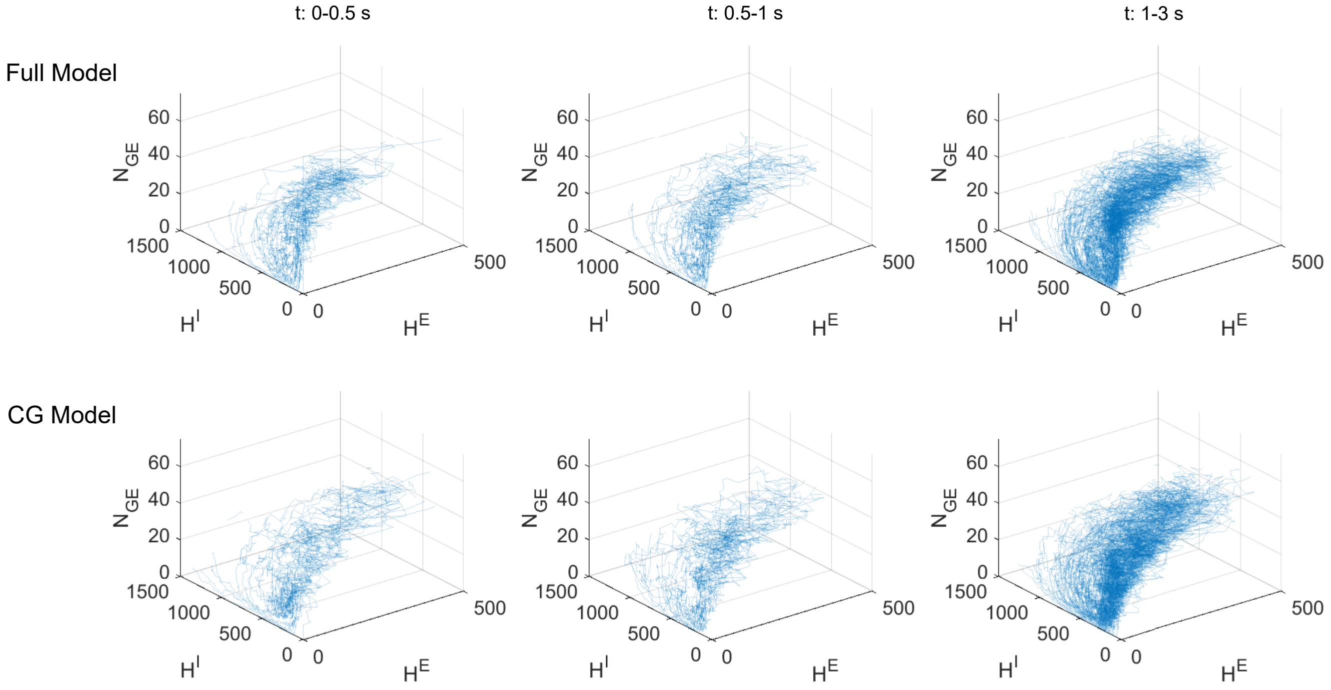

Figure S3. Full and CG model trajectories in different time period shown in the 3D subspace (N_{GE}, H^E, H^I). **Upper:** The trajectories of MIF model in different time period; **Lower:** The trajectories of CG model in different time period.

Convergence of Entropy. To demonstrate the temporal stability of entropy computed in Fig. 8, we here present entropy computed from 1/3/5/10-second simulations separately and the standard error from 10-second simulations. All entropy exhibit convergence after 3s, and the standard error (SE) stay low as $O(10^{-2})$.

Entropy	Syn	Reg	Hom	Tra4	Tra5	Tra6	Tra7	Tra8	Tra9
1s	11.28	11.85	12.53	10.33	11.40	11.43	11.62	11.97	11.80
3s	11.70	12.38	13.27	10.72	11.91	11.80	12.26	12.66	12.43
5s	11.77	12.51	13.45	10.86	11.98	11.97	12.48	12.83	12.59
10s	11.77	12.58	13.52	10.88	12.01	11.92	12.63	12.94	12.68
SE of 10s	0.04	0.06	0.05	0.04	0.06	0.06	0.02	0.04	0.02

Table S1. Entropy computed in different time intervals for all regimes investigated in Fig. 8.

2 A LARGER MIF NETWORK

We repeat the model reduction for a 400-neuron MIF network, and collect the state space variables $(N_{GE}, N_{GI}, H^E, H^I)$ from a 100-second simulation (Fig. S4). A similar manifold is revealed by the trajectories in the state space, and also verified by the mass of trajectory density (Fig. S4AB). The two-dimensional local structure is also verified by the local dimensionality estimation and local linear embedding (Fig. S4CD). For this network, we simulate the Syn regime with parameters identical to Li et al, 2019.

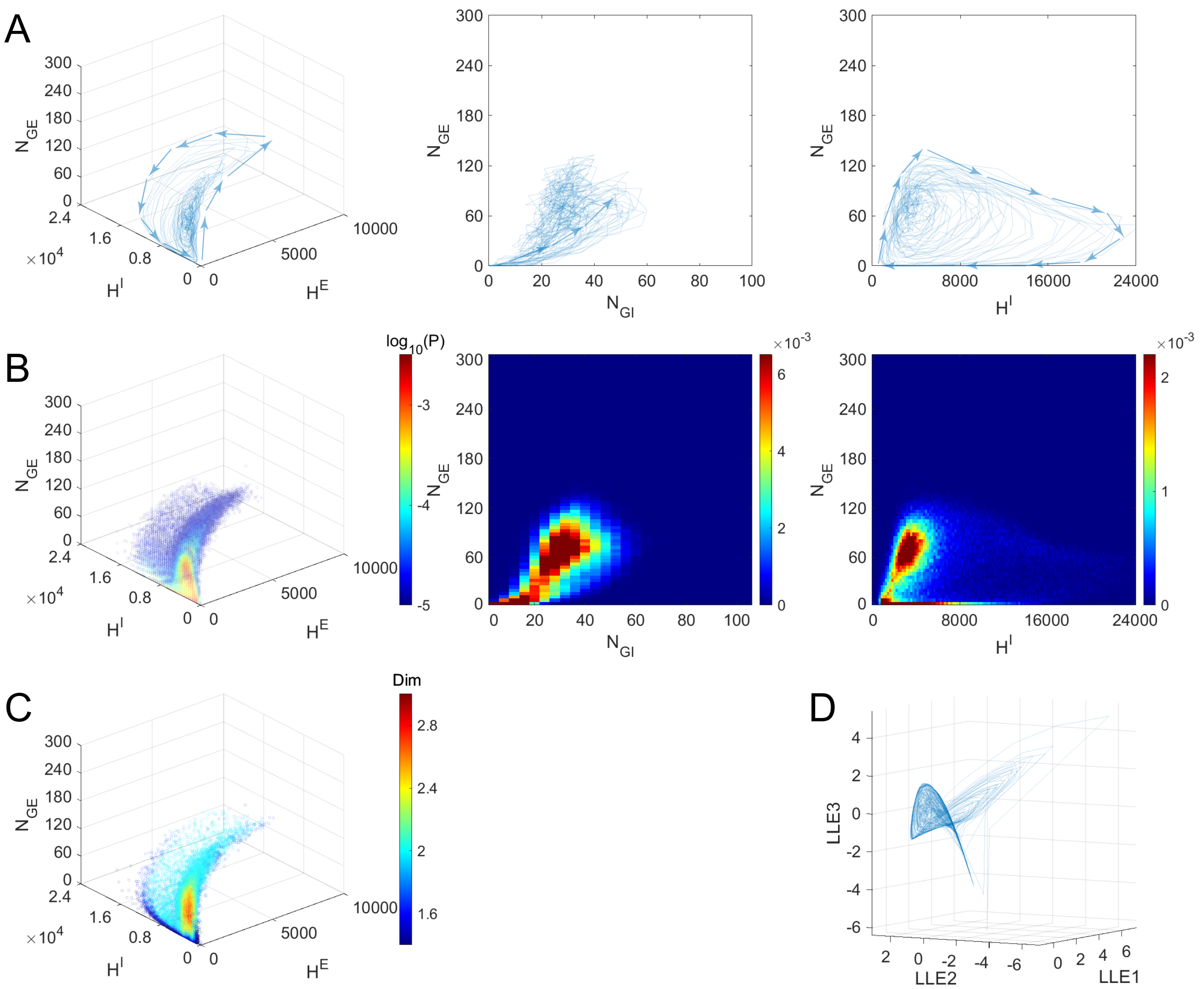

Figure S4. Gamma dynamics of a 400-neuron MIF network restricted on a low-dimensional manifold. **A-D** are similar to **A, B, E, F** of Fig. 6.

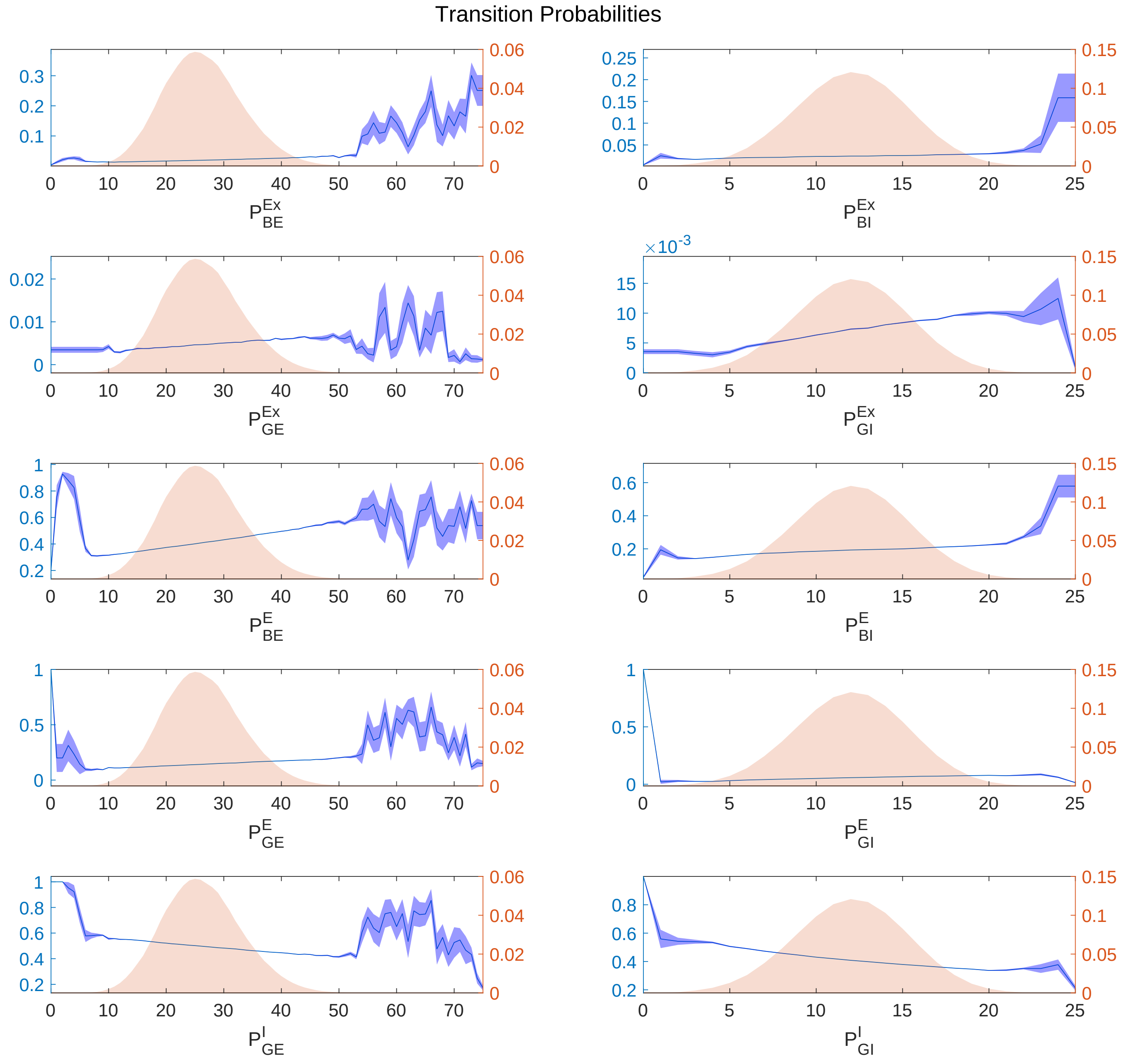

Figure S5. Estimations of all transfer probabilities. **Blue:** Transition probabilities and their standard errors obtained from 30-second simulations; **Light red:** Marginal distributions of the states from full model simulations, depending on N_{GE} or N_{GI}.

3 ISSUES OF MODEL REDUCTION

Reliability of transition probabilities. The performances of reduced models delicately depend on the reliability of transfer probabilities, which are estimated from full model simulations. In Fig. S5, we present all transfer probabilities and their standard errors as functions of N_{GE} or N_{GI}, which are estimated from 30-second full model simulations (blue). Meanwhile, we use marginal distributions of the states from full model simulations depending on N_{GE} or N_{GI} as references (light red). Here we choose $\tau_{ee} = 2.5$ ms. As shown below, the reliability of transition probabilities is indicated by the small standard errors ($o(10^{-2})$) in the states where the majority mass of marginal probabilities lies. On the other hand, high estimation errors are involved for extreme values of N_{GE} and N_{GI} due to the rarity of corresponding events sampled from the full model simulations.

Reusing full model simulations. The full model simulation of one regime can be reused for other regimes to generate new transition probabilities as long as they share similar geometrical information of the subthreshold distributions. For example, in Fig. S6, we only simulate the full model once for $\tau^{EE} = 2.5$ ms and keep using the results for all other parameter sets to compute spike synchrony indexes. Compared to Fig. 5B in the main text, where we use different full model simulations for all τ^{EE}s, reusing simulations exhibits a similar capacity of capturing statistics.

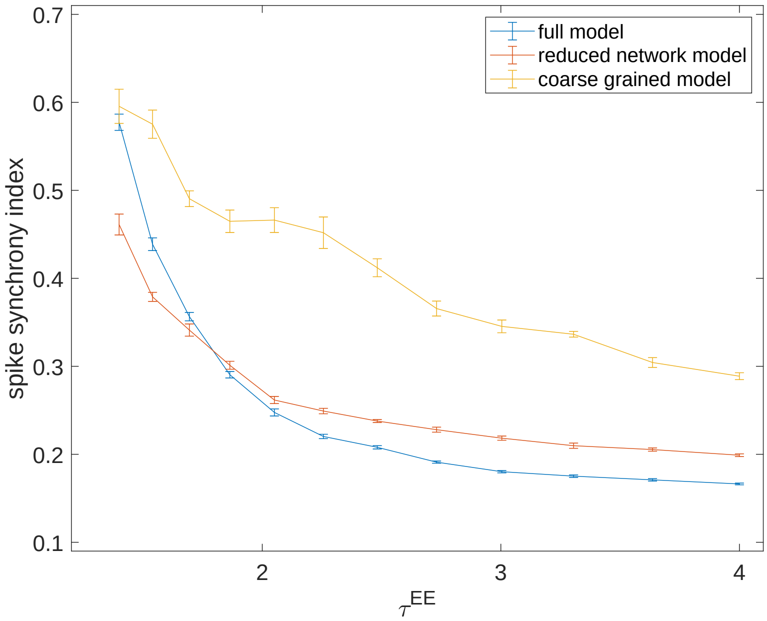

Figure S6. Degree of synchrony versus τ^{EE} in three models. Similar to Fig. 5B, but here we keep using full model simulations for $\tau^{EE} = 2.5$ ms.

Firing rates in three models. Our reduced models faithfully capture network firing rates in different parameter regimes. In Fig. S7, we compare the firing rates of three models when varying τ^{EE} (upper) and external input rates (lower).

Selection of the cutoff. The faithfulness of reduced models depends on the selection of cutoff V^c for gate/base neurons. Recall our assumption that the gamma dynamics are primarily sensitive to the number of gate neurons. Therefore, a high V^c provides an accurate description of gate neurons, but roughly for the base neurons, and vice versa. This could be a potential issue for the RN model. In Fig. S8, we present the raster plots (left) and one transfer probability (P^E_{BE}, right) for different selections of cutoff V^c in the RN model (Syn regime). The transfer probability changes slowly with cutoff values. On the other hand, different cutoff choices impact the synchronicity and firing rates according to the raster plots. We choose $V^c = 45$ throughout the whole paper (red box) as a compromise.

The duration of MFEs. The MFEs of the reduced models have longer duration than the full model, possibly due to the slower probability flows since we combine multiple states from the full model as one in the reduced ones (Fig. S9).

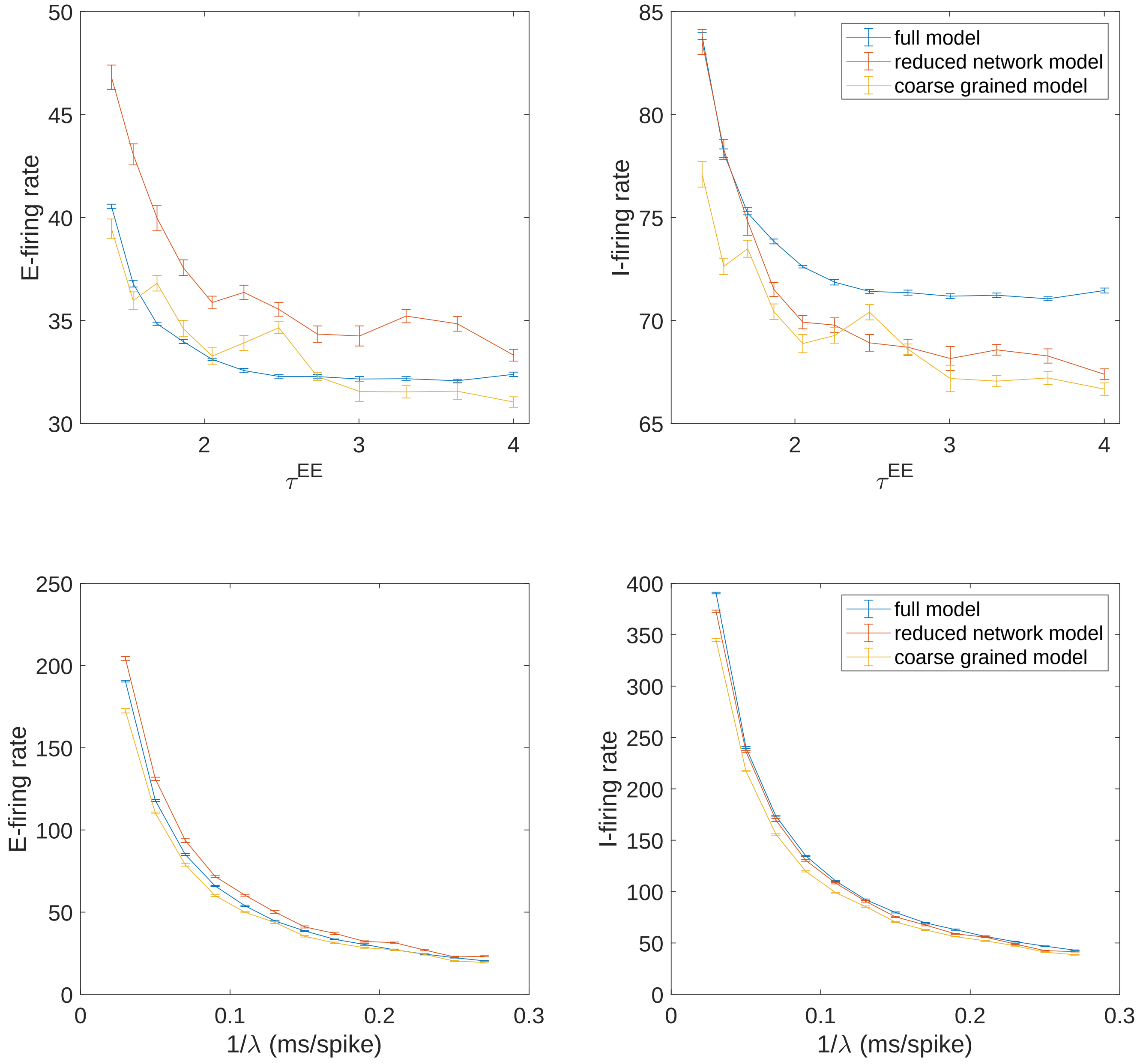

Figure S7. Upper: E/I-Firing rates of three models versus τ^{EE}; **Lower:** E/I-Firing rates of three models versus external input rates, indicated by $1/\lambda$.

A 5-variable CG model. The 4-variable CG model combines E-kick pools of all neurons together, which may cause a problem since the pending E-kicks for E neurons and I neurons are consumed by different speeds. Hence we come up with a 5-variable version were H_{EE} and H_{IE} are counted separately (Fig. S10).

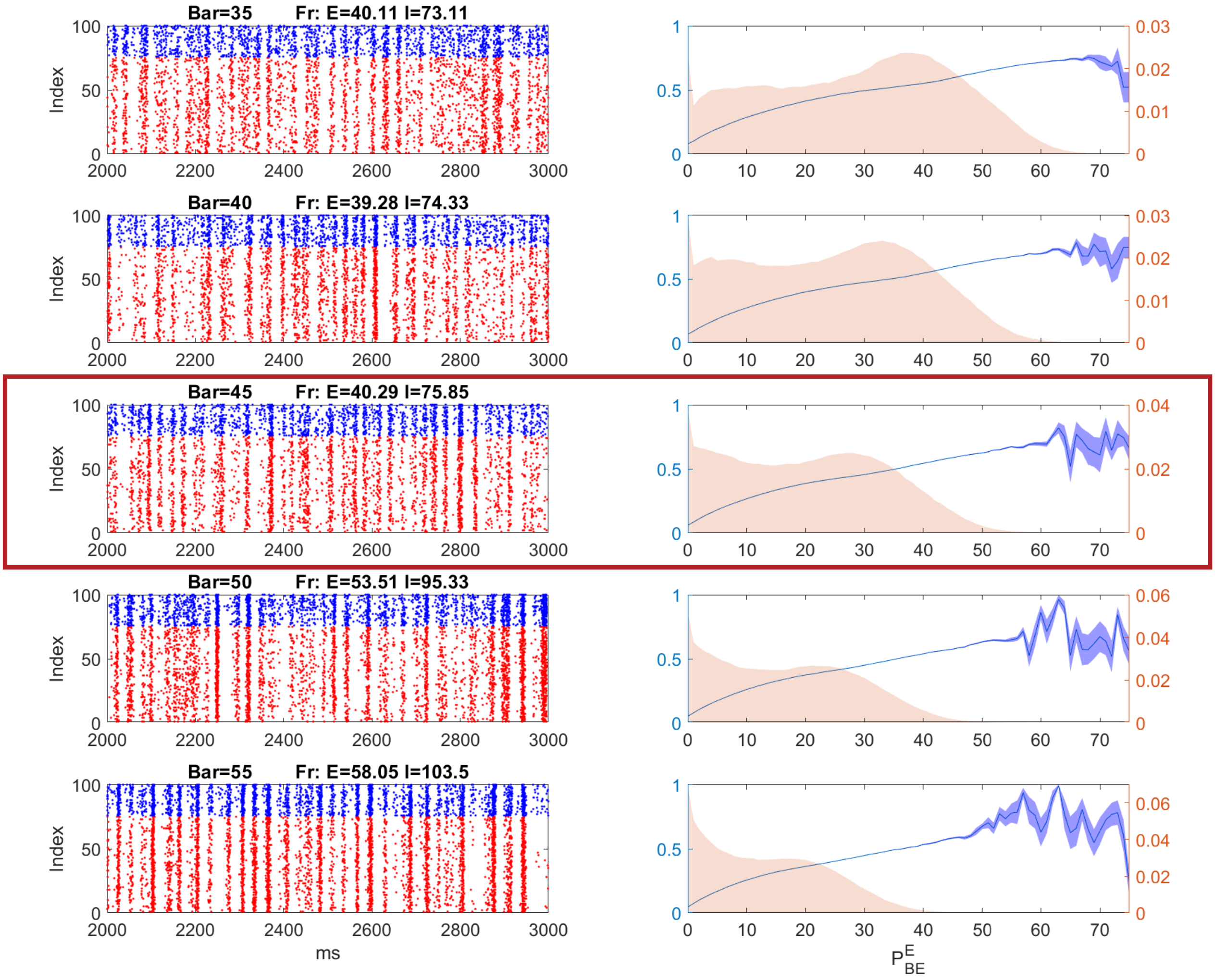

Figure S8. Different choices of cutoff have an impact on RN model dynamics. In the Syn regime, we vary V^c from 35 to 55. **Left:** Raster plots with firing rates indicated in titles. **Right:** Estimations of P^E_{BE} (blue, with standard errors), and marginal distributions of states sampled from the full model simulations (light red).

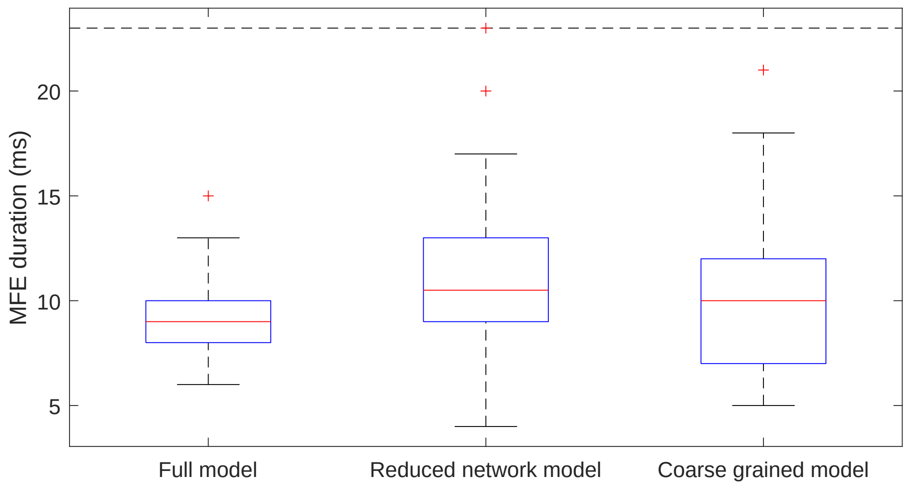

Figure S9. The duration of MFEs in different models. For each model, the mean (red line), first and third quartile (lower and upper bounds of the blue box), minimum and maximum (black lines), and outliers (red cross) are indicated in the box plot. The duration of MFEs for the RN and CG models are significantly higher than the full model (p = 0.004 and 0.038).

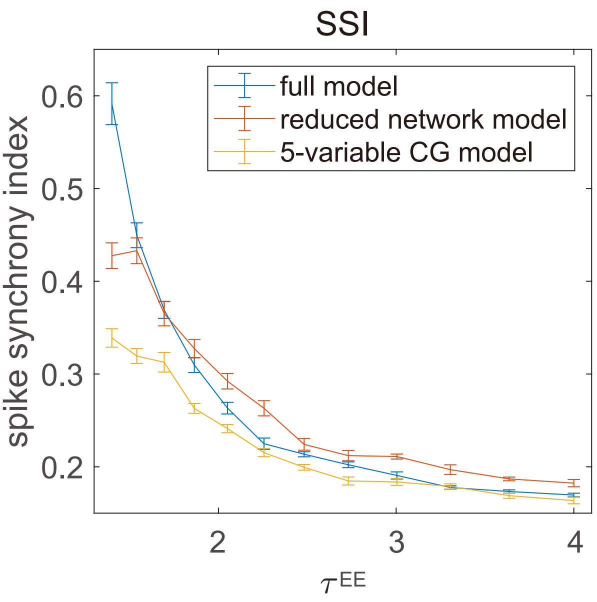

Figure S10. Degree of synchrony increases when τ^{EE} decreases. Reprinted from Fig. 5B, but the 4-variable CG model is replaced by a 5-variable version.
